# Index and biological spectrum of human DNase I hypersensitive sites

**DOI:** 10.1038/s41586-020-2559-3

**Published:** 2020-07-29

**Authors:** Wouter Meuleman, Alexander Muratov, Eric Rynes, Jessica Halow, Kristen Lee, Daniel Bates, Morgan Diegel, Douglas Dunn, Fidencio Neri, Athanasios Teodosiadis, Alex Reynolds, Eric Haugen, Jemma Nelson, Audra Johnson, Mark Frerker, Michael Buckley, Richard Sandstrom, Jeff Vierstra, Rajinder Kaul, John Stamatoyannopoulos

**Affiliations:** 1 Altius Institute for Biomedical Sciences, Seattle, WA USA; 2 Department of Genome Sciences, University of Washington, Seattle, WA USA; 3 Division of Oncology, Department of Medicine, University of Washington, Seattle, WA USA

**Keywords:** Data integration, Epigenomics, Functional genomics, Epigenomics

## Abstract

DNase I hypersensitive sites (DHSs) are generic markers of regulatory DNA^1–5^ and contain genetic variations associated with diseases and phenotypic traits^6–8^. We created high-resolution maps of DHSs from 733 human biosamples encompassing 438 cell and tissue types and states, and integrated these to delineate and numerically index approximately 3.6 million DHSs within the human genome sequence, providing a common coordinate system for regulatory DNA. Here we show that these maps highly resolve the *cis*-regulatory compartment of the human genome, which encodes unexpectedly diverse cell- and tissue-selective regulatory programs at very high density. These programs can be captured comprehensively by a simple vocabulary that enables the assignment to each DHS of a regulatory barcode that encapsulates its tissue manifestations, and global annotation of protein-coding and non-coding RNA genes in a manner orthogonal to gene expression. Finally, we show that sharply resolved DHSs markedly enhance the genetic association and heritability signals of diseases and traits. Rather than being confined to a small number of distal elements or promoters, we find that genetic signals converge on congruently regulated sets of DHSs that decorate entire gene bodies. Together, our results create a universal, extensible coordinate system and vocabulary for human regulatory DNA marked by DHSs, and provide a new global perspective on the architecture of human gene regulation.

## Abstract (web summary)

High-resolution maps of DNase I hypersensitive sites from 733 human biosamples are used to identify and index regulatory DNA within the human genome.

## Main

A fundamental challenge in modern biology is to delineate with the highest possible precision the repertoire of regulatory DNA elements encoded within the human genome sequence. A universal feature of active *cis*-regulatory elements—promoters, enhancers, silencers, chromatin insulators or enhancer blockers, and locus control regions—is focal alteration in chromatin structure triggered by binding of transcription factors (TFs), which supplants a canonical nucleosome and renders the underlying DNA accessible to nucleases and other protein factors^1,9^. For more than 40 years^2,10,11^, DHSs have provided reliable signposts for high-precision delineation of regulatory DNA in complex genomes^1–5^. DHSs typically mark compact (less than 250 base pair (bp)) elements, and their appearance over a *cis*-regulatory region signifies its actuation (readying for activation), which may occur before, or coincident with, its functional activation. DHS mapping thus provides a generic tool for illuminating both active and potential regulatory landscapes.

The advent of genome-scale mapping of DHSs^12–15^ and its application to diverse human and mouse cell and tissue types^16,17^ has yielded many insights into the organization^16^, evolution^17–19^, activity^15,16,20^, and function^16,21,22^ of human regulatory DNA in both normal and malignant states^23^. A cardinal property of regulatory DNA is that its accessibility is cell type- and state-selective, with only a small fraction of all genome-encoded elements becoming actuated in a given cellular context^16,23^.

The overwhelming majority of disease- and trait-associated variants identified by genome-wide association studies (GWASs) lie in non-coding regions of the genome, and these variants are most strongly enriched in DHSs mapped in disease-relevant cell contexts^6,7^. DHSs also collectively contain the GWAS variants that account for the majority of trait heritability explained by genotyped single-nucleotide polymorphisms (SNPs)^8^. Deeper insights into the connection between GWAS variants and gene regulation have been limited by the lack of comprehensive annotations that capture the biological behaviour of regulatory DNA.

As genome-scale data from diverse cellular contexts have accumulated, systematic annotation of cell type- and state-selective DHSs has grown increasingly challenging, and it has also become evident that large sets of DHSs distributed widely across the genome may share common regulatory programs^16^. However, the annotation and analysis of state-selective behaviours has been hampered by the lack of a common coordinate system for DHSs.

Here we sought to expand the breadth of high-quality DHS maps, and to unify them into a common reference framework that achieves precise genomic annotation by integrating observed biological variability in the manifestation of accessibility at individual elements, and that captures complex cell-selective behaviours in a quantitative fashion. We report a coherent framework and demonstrate its utility for the annotation of human regulatory DNA and gene landscapes; for defining how regulatory programs are encoded within the genome; and for clarifying links between genetic signals and gene regulation to enable new insights into the organization and interpretation of non-coding variation associated with diseases and traits.

## Index of consensus human DHSs

**Fig. 1:**
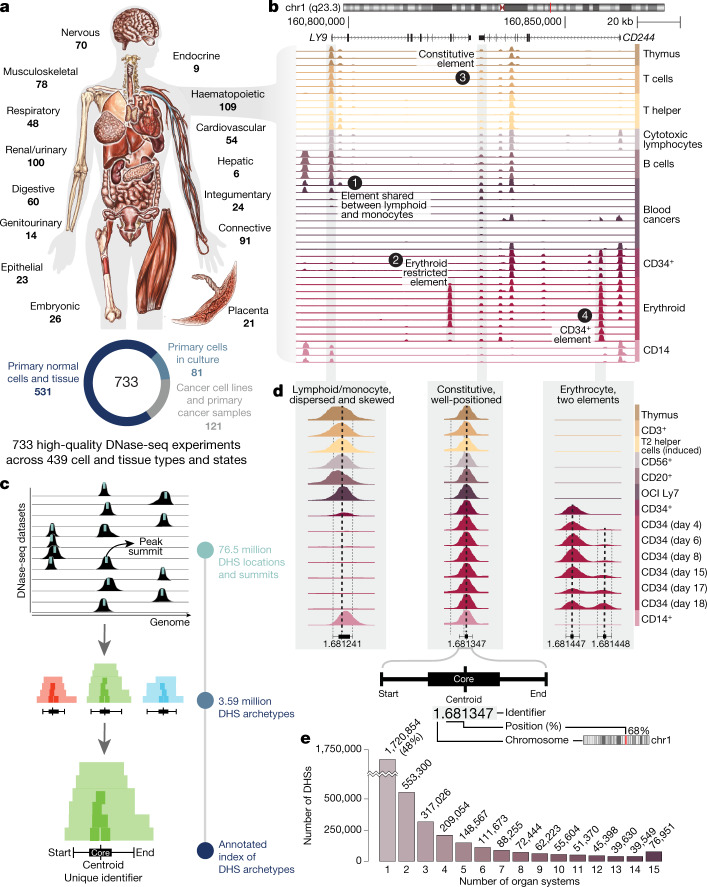
Index of DHSs in the human genome. **a**, DNA accessibility assayed across multiple biosamples (indicated) from the main human organ systems. Of 733 biosamples, 531 were derived from primary cells and tissues. **b**, Example locus on chromosome 1, showing DNase I cleavage density in haematopoietic biosamples (right) with cell type-selective differences. **c**, Outline of DHS index procedure; 76.5 million DHSs aggregated across individual datasets jointly delineate and annotate 3.59 million consensus DHSs. **d**, Examples of consensus DHSs with varying cell-type selectivity and genome positional stability. Annotations include consensus DHS coordinates (start/end), single-base ‘centroid’, ‘core’ region aggregating centroids across biosamples, and a unique numerical identifier. **e**, Number of organ systems across which DHSs are shared.

**Extended Data Fig. 1:**
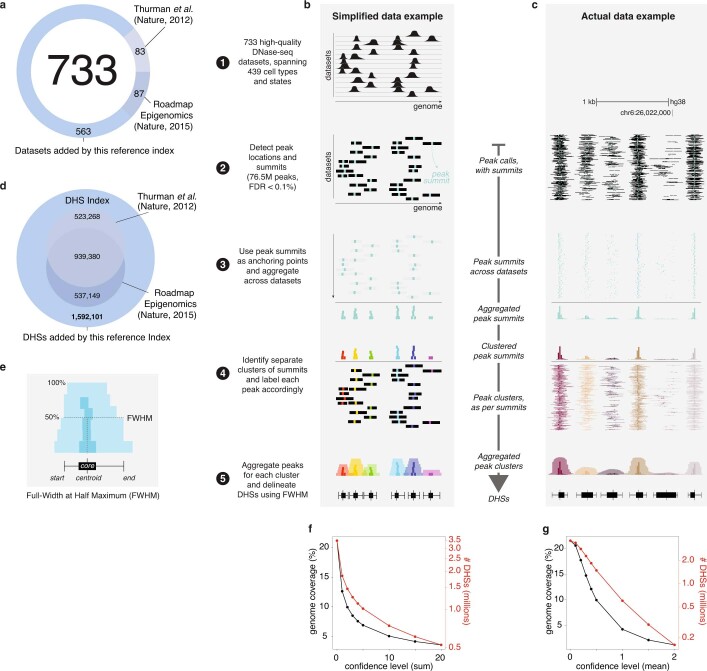
Construction of a DHS index. **a**, Increase in number of DNase-seq datasets relative to previous efforts. **b**, **c**, Delineation of index DNase I hypersensitive sites (DHSs) from raw DNase-seq signal tracks, shown for simplified data (**b**) and actual data (**c**). Starting from individual DNase-seq datasets (step 1), we call peaks in each dataset (step 2), aggregate peak summits into clusters, indicating isolated accessibility events (step 3), group full peak coordinates according to these clusters (step 4), and delineate DHSs using full-width at half maximum (FWHM) (step 5). **d**, Increase in number of detected DHSs relative to previous efforts. **e**. Detailed view of FWHM delineation. **f**, **g**, Confidence scores based on DNase I signal strengths assigned to each DHS, allowing for pragmatic filtering using either summed (**f**) or mean (**g**) signal strength—the former assigning high confidence scores to DHSs with overall high signal levels across datasets, the latter providing a score normalized by the number of datasets in which a DHS was observed.

To create deeply sampled reference maps of human regulatory DNA marked by DHSs, we performed DNase I hypersensitive site sequencing (DNase-seq)^15^ on a wide range of human cell and tissue biosamples that span all major human organ systems (Fig. 1a). Reference-grade data were created by rigorous quality screening for complex libraries yielding high signal-to-noise ratio data (Methods), and were aggregated with prior high-quality data from the ENCODE^16^ and Roadmap Epigenomics^24^ projects. We conservatively selected 733 biosamples that represent 438 cell or tissue types and states (Fig. 1a, Supplementary Table 1, Methods), the majority of which were derived from primary ex vivo cells and tissues (72% of samples) or from primary cells in culture (11%), with the remainder (17%) from immortalized cell lines. Collectively these samples represent an approximately 5.5-fold expansion of sampled cell and tissue types and states relative to the previous phase of ENCODE^16^ (Extended Data Fig. 1a), and the resulting data reveal rich and varied patterns of DNase I hypersensitivity (Fig. 1b).

## Common coordinates for regulatory DNA

We sought to create a precise and durable common reference framework for genomic elements that encode DHSs by (i) comprehensively and stringently (0.1% false discovery rate (FDR)) delineating DHSs within each biosample; (ii) integrating individual biosample DHS maps to define archetypal DHS-encoding sequence elements within the human genome sequence; and (iii) assigning to each archetypal element a unique numerical identifier (Fig. 1c).

We identified an average of 104,433 DHSs per biosample, and a total of 76,549,656 DHSs across all 733 biosamples. To delineate archetypal DHS-encoding genomic elements, we developed the consensus approach outlined in Fig. 1c and Extended Data Fig. 1b, c. First, we computed the summit coordinate (1 bp) of each DHS peak and aligned these across all biosamples to define a consensus centroid for each archetypal DHS. To resolve DHS boundaries, we collated the local linear extent of DNase I hypersensitivity into a consensus range (Methods). We then combined centroids and boundaries into a single index of 3,591,898 spatially distinct DHS-encoding sequence elements, greatly eclipsing both the number (approximately 2.5-fold) and precision of DHSs delineated during earlier phases of ENCODE (Extended Data Fig. 1d). In addition to a consensus summit (centroid) and start and end coordinates, each archetypal DHS additionally comprises a ‘core’ region that represents empirical confidence bounds on the centroid (Fig. 1d, Extended Data Fig. 1e). Each archetypal DHS derives from an average of 21 biosamples, and because each DHS from a given biosample contributes to a single archetypal DHS, the provenance of each DHS in the index can be directly traced back to its contributing biosample(s).

Finally, we assigned a unique identifier to each archetypal index DHS using a numerical schema (Fig. 1d) that (i) conveys the genomic localization of each DHS; (ii) enables unlimited extension to newly discovered elements; (iii) ensures compatibility with future reference genome builds and portability to personal genomes; and (iv) enables direct integration with DNase I footprints^25^ or other experimental annotations (Methods). We also assigned confidence scores to all index DHSs that combine signal strength with propensity for repeated observation in independent biosamples (Extended Data Fig. 1f, g).

**Extended Data Fig. 2:**
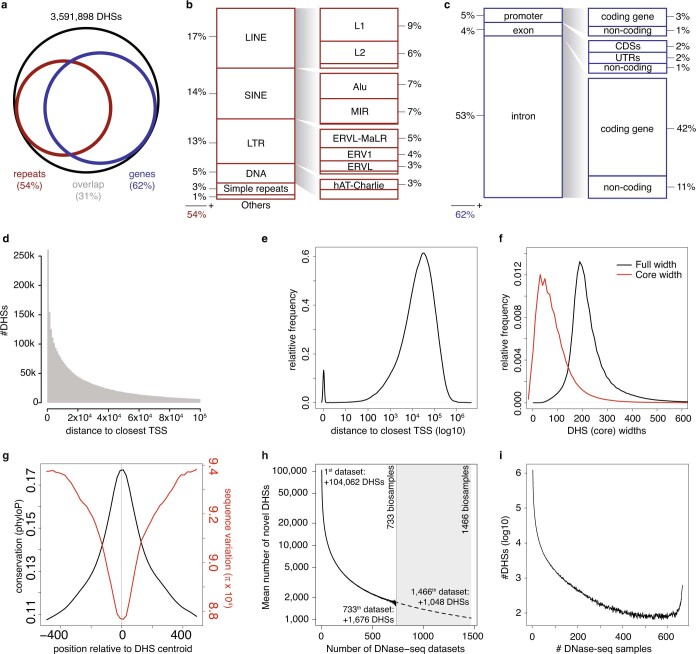
Genomic context of DHS index elements. **a**, Overall coverage of 3.5M+ DHSs across genes and repetitive elements. **b**, Coverage of classes and families of repetitive elements. **c**, Coverage of annotated genic regions. **d**, Barplot of the number of DHSs as a function of distance to the nearest annotated transcription start site (TSS), up to 100,000 base pairs. **e**, Density plot of DHS distance to the closest TSS for all index DHSs, showing that the vast majority of DHSs are found distal to annotated promoters. **f**, Density plot of element widths for full DHSs and their core regions only, shown for DHSs observed in more than one biosample. Uniform 20 bp jitter added for smoothness. **g**, DHS centroids show an increase in sequence conservation (phyloP) and a decrease in within-human sequence variation (TOPMed, *π* × 10^4^). **h**, Mean number of new DHSs observed as a function of the *n*th DNase-seq dataset added, shown for the first 733 observed biosamples, as well as for an extrapolation to an additional future 733 new biosamples. **i**, Histogram indicating the variety in cell type selectivity of DHSs, ranging from single cell types to groups of 10s, 100s or even all assayed cellular conditions.

Index DHSs are broadly distributed across annotated genic and repetitive elements (Extended Data Fig. 2a–e). Fifty-three per cent of DHSs lie within introns, about 3% within non-coding exons and untranslated regions (UTRs), and about 2% are dually encoded within protein-coding exons (Extended Data Fig. 2c). Although DHSs are pronounced at annotated transcription start sites (TSSs), most localize to regions away from TSSs (Extended Data Fig. 2d, e). Fifty-four per cent of index DHSs overlap repetitive elements of all classes and subfamilies (Extended Data Fig. 2b), consistent with previous observations^26^, although overlap with DHS cores (41%) and centroids (37%) suggests that a more focused subset of DHSs derives regulatory machinery from repeats.

## Proportion of the genome that encodes DHSs

The full extent ot the DHS landscape should define—or at least closely approximate—the canonical *cis*-regulatory compartment of the genome, the size of which has been the subject of considerable debate^27^. The roughly 3.6 million consensus DHSs have an average width of 204 bp (median 196 bp, interquartile range (IQR) 151–240 bp) and collectively span 665.57 Mb (21.55%) of the reference human genome sequence. DHS cores have an average width of 55 bp (median 38 bp) (Extended Data Fig. 2f) and span 197.74 Mb (6.4%) of the genome. DHS centroids also precisely mark the peak in evolutionarily conserved nucleotides within DHSs, and the corresponding trough in the average density of human genetic variants (Extended Data Fig. 2g), which are discontinuously distributed between TF-occupied and unoccupied subsegments of DHSs^25^. Iterative subsampling of the 733 biosamples showed that the addition of any given new biosample would be expected to contribute about 1,676 new DHSs to the index (median 283, range 4–64,054, 95% confidence interval 1,344–2,009) (Extended Data Fig. 2h and Methods). Extrapolating from this, the addition of an additional biosample collection of equivalent size (*n* = 733) would be expected to increase annotated DHS elements by about 27% (Extended Data Fig. 2h). Notably, increasing biosamples should increase the precision of annotation and thus the resolution of some broader elements (such as Extended Data Fig. 1c, second DHS from the right) to two or more distinct archetypal DHSs; however, such elements are in the minority.

## Cellular patterning of DNA accessibility

**Fig. 2:**
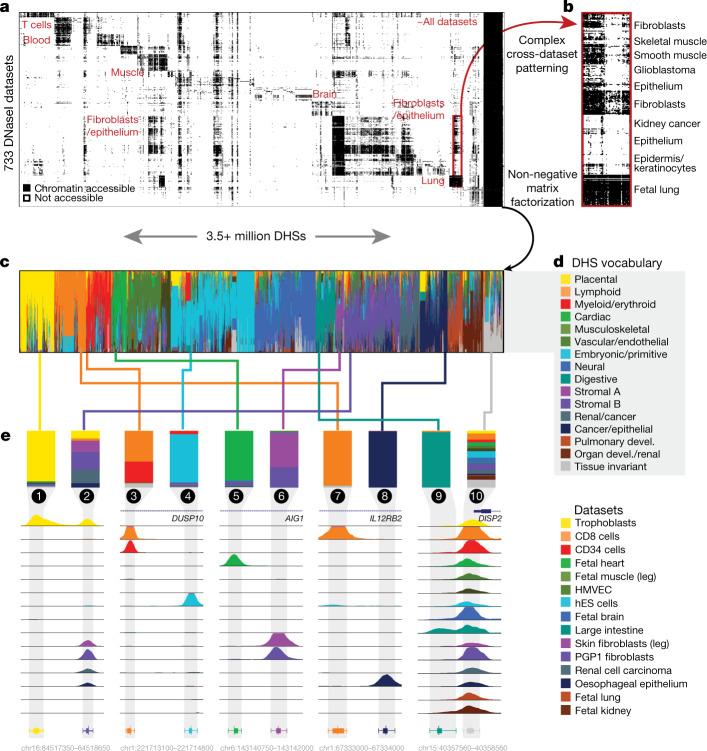
A simple vocabulary captures complex patterning of DHSs. **a**, DNA accessibility at 3.59 million consensus DHSs assayed across 733 biosamples encapsulated in a visually compressed DHS-by-biosample matrix. Recurring accessibility patterns indicate extensive sharing across cell contexts. Dark column (right) shows DHSs detected in (nearly) all datasets. **b**, Modular behaviour of DHS actuation illustrated by thousands of DHSs with similar cross-biosample accessibility patterns. **c**, Decomposition of DHS actuation patterns across 733 biosamples into 16 components using NMF. The cellular patterning of each DHS is described using a mixture of components, indicated by distinct colours. **d**, DHS component labels provide a regulatory vocabulary for DHSs. **e**, Component mixtures for ten example DHSs with varying degrees of component specificity. The biosample dataset most strongly associated with each component is shown. Bottom, annotation of individual DHSs with a single dominant component.

DHSs are extensively shared across both individual biosamples and groups of biosamples from different organ systems (Fig. 1e, Extended Data Fig. 2i). It was previously reported that groups of widely distributed DHSs with closely shared cross-cell-type actuation patterns also shared biological functions such as enhancer activity^16^. Patterns of index DHS actuation across the 733 biosamples (Fig. 2a) were complex, with both highly modular and less coherent structures (Fig. 2b). The majority of DHSs showed complex actuation patterns rather than simple cell-selective behaviour (Fig. 1e, Extended Data Fig. 2i), prompting us to develop a flexible approach for quantifying and annotating these patterns.

In principle, the actuation of any given index DHS across cell states can be summarized by a limited number of biological ‘components’ combined in a weighted fashion. Orthogonally, the same components can be used to summarize the DHS repertoire of an individual biosample. Because DHS-centric information can inform biosamples and vice versa, a key advantage of this approach is its potential to capture complex behaviours while providing biological interpretability.

## A vocabulary for regulatory patterns

**Extended Data Fig. 3:**
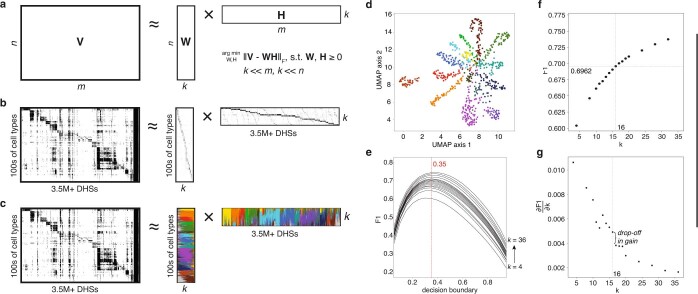
NMF decomposition of DHS index. **a**, Schematic of non-negative matrix factorization (NMF) applied to an *n*-by-*m* matrix resulting in *k* components. The objective is to minimize the difference between the original matrix **(V)** and the product of **(W)** and **(H)**, such that all elements of **(W)** and **(H)** are non-negative. **b**, Depiction of NMF applied to our DNase-seq dataset of 733 biosample datasets and 3.5M+ DHSs, using *k* components. **c**, Colour-based view from the values shown in **b**. Colours indicate relative loadings of each NMF component, for both biosamples and DHSs. **d**, Two-dimensional UMAP projection of 733 biosamples coloured by their strongest representative NMF component. **e**, Choice of NMF decision boundary (0.35) based on maximal F1 score as a function of number of components *k* (4 to 36). **f**, F1 score as a function of the number of components *k*, with the chosen *k* = 16 and corresponding F1 score indicated. **g**, Gradient showing reduced gain in F1 score after *k* = 16.

To simplify the matrix of 3,591,898 DHSs × 733 biosamples we applied non-negative matrix factorization^28^ (NMF) (Extended Data Fig. 3a–d), a technique that was initially used in the field of computer vision for learning parts-based representations of objects and semantic features of text^29^. We represented each DHS by a large enough number of components (*k* = 16) to ensure accuracy—that is, the degree to which the original matrix can be reconstructed from the components—while retaining potential for interpretability via assignment of components to established biological contexts such as known cell lineage relationships, or cell states known to be specified by specific regulatory factors (Fig. 2c, Extended Data Fig. 3e–g, Methods).

**Extended Data Fig. 4:**
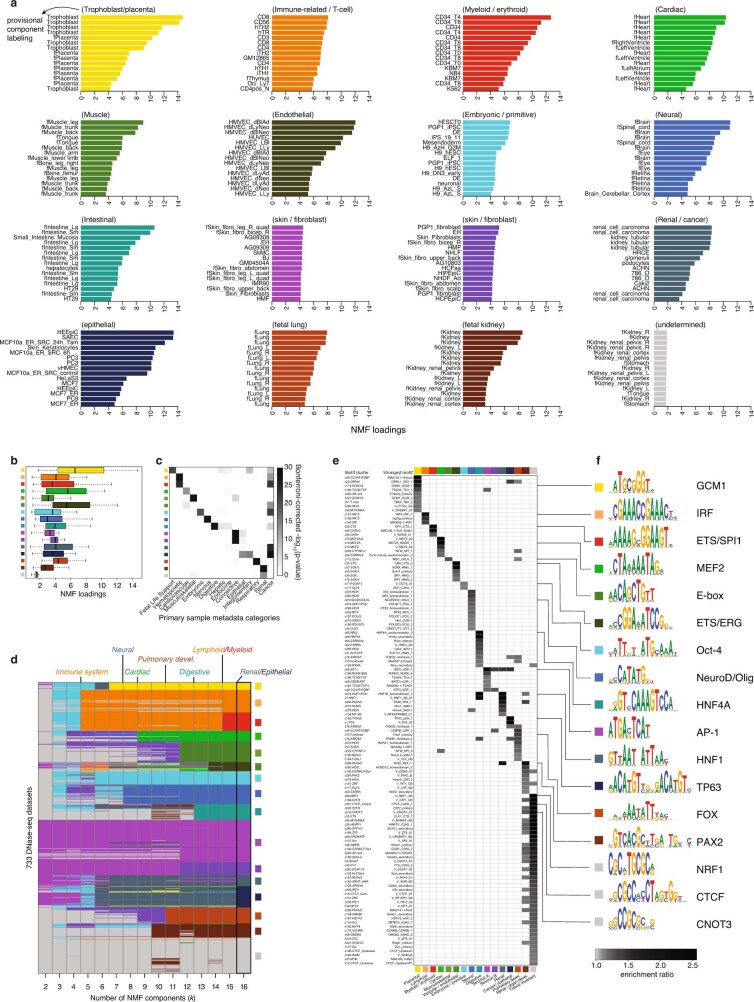
Association of DHS components with cellular conditions and TF motifs. **a**, Bar plots showing for each NMF component the top 15 DNase-seq datasets in terms of NMF loadings. NMF loading strength (*x*-axes) and dataset labelling (*y*-axes) are indicated. **b**, Box plots showing for each NMF component its loadings across those biosamples for which that component is maximally loaded. Boxes denote medians and interquartile ranges (IQRs, 25–75%), whiskers represent 1.5 × IQRs, *n* = 18,57,46,27,52,23,34,49,40,107,33,27,54,40,36,90 biosamples, respectively. **c**, Beyond the top 15 biosamples for each component, general associations of components with annotations regarding human organ systems and cancer. Indicated are Bonferroni corrected *P* values, resulting from one-sided Mann–Whitney U tests. **d**, Distribution of biosamples across (maximal) NMF components, for the number of components (*k*) ranging from 2 to 16. Labels at the top indicate at which point distinct lineages became represented in corresponding components. **e**, Enrichment of transcription factor (TF) binding motifs in DHS components. Greyscale values indicate enrichment levels, only statistically significant results are included. DHS components shown on the *x*-axis, TF motif clusters with top representative motif on the *y*-axis. **f**, Top enriched TF motifs for each DHS component.

**Extended Data Fig. 5:**
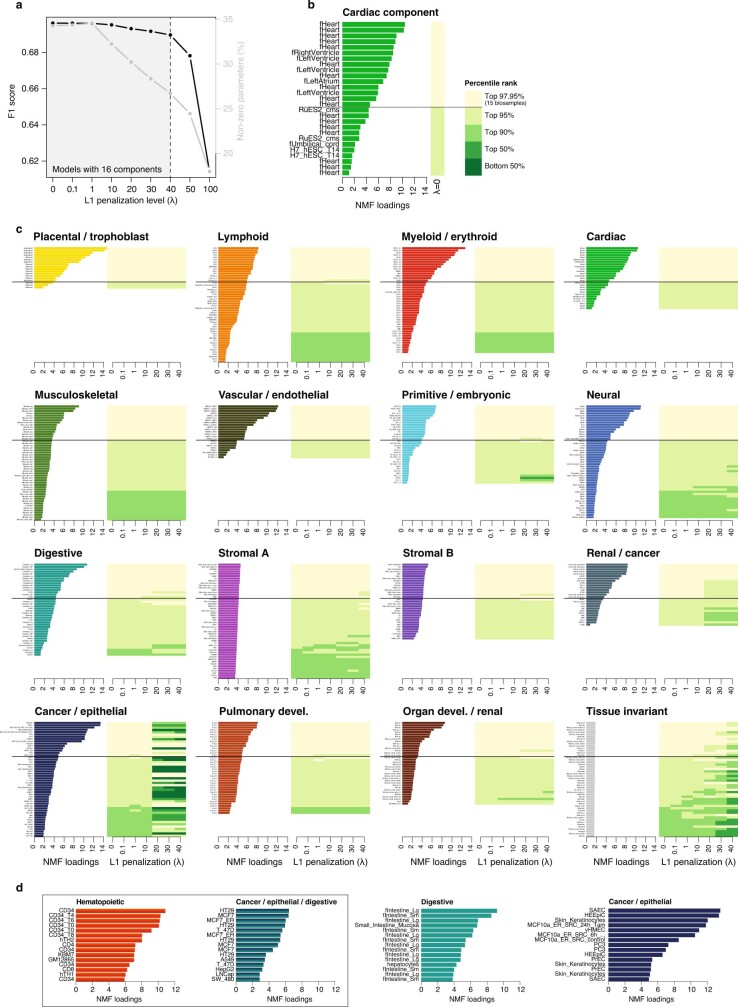
DHS component robustness. **a**, F1 score as a function of L1 penalization levels (ƛ), with separately indicated levels of sparsity reflected by the percentage of non-zero parameters in the resulting models. Shaded area represents penalization levels resulting in comparable 16-component models, as opposed to models with effectively less than 16 components, which are discarded in subsequent analyses. **b**, All biosamples with non-zero NMF loadings in the cardiac DHS component (for ƛ = 0). Horizontal line separates the top 15 biosamples (yellow shading) from the rest (shades of green), where green shading indicates quantile ranking in terms of component loading strength. **c**, Biosamples with non-zero NMF loadings for each DHS component, extended with agreement of quantile ranking as a function of L1 penalization levels, indicating that these rankings stay near constant for most components. **d**, Top 15 biosamples in terms of NMF loading per DHS component for an alternative NMF model resulting from a 40% downsampling of high-quality haematopoietic biosamples. NMF loading strength (*x*-axes) and dataset labelling (*y*-axes) are indicated, only for components that differ with the final model.

To connect components with biological contexts, we identified the biosamples that were most strongly associated with each component, and the distribution of TF recognition sequences within DHSs that was most strongly associated with that component. For all components, the top contributing cell or tissue samples were notably coherent, enabling provisional assignment of a meaningful biological label to most components (Extended Data Fig. 4a–d, Methods). Enrichment of TF recognition sequences within the DHSs that were most strongly associated with each component revealed clear mappings between distinct sets of cell lineage- or state-specifying TFs and specific components (Extended Data Fig. 4e, f, Methods), orthogonal to the biosample-to-component mappings described above. Finally, we combined biosample-to-component mappings and TF-to-component mappings to create a regulatory ‘vocabulary’ that captures the actuation pattern of a DHS across cell types and states (Fig. 2d, Supplementary Note). Notably, the interpretation of components does not change with further induced model sparsity (Extended Data Fig. 5a–c) or changes in cell type representation (Extended Data Fig. 5d).

## Biological annotation of individual DHSs

We next sought to annotate each DHS with a regulatory ‘barcode’ that captures its tissue manifestations. The cross-biosample actuation pattern of each DHS is captured by linear combinations of NMF components (Fig. 2c, Extended Data Fig. 3a–c), providing a de facto barcode of its biological spectrum (Fig. 2c, e). DHSs that are selective for a single cell type or state are annotated by a single majority component (Fig. 2e, columns 1, 4, 5, 7–9); DHSs that occur in multiple cellular contexts are described by combinations of components (Fig. 2e, columns 2, 3, 6, 10); and constitutive DHSs are annotated by mixtures of all components (Fig. 2e, column 10), including a component that describes tissue-invariant behaviour. In this schema, DHSs with similar cross-biosample actuation patterns exhibit similar mixtures of components. For analytical practicality and visual compactness, the annotation of each DHS can be further summarized using its strongest single component (Fig. 2e, bottom); we use this summary vocabulary for the analyses described below.

## Dense encoding of regulatory information

The above results indicate that DHSs have the potential for surprisingly diverse biological regulatory patterns that combine coordinated positive (actuation) and negative (quiescent) behaviours. As the overwhelming majority of DHSs fall into a tight size range that stays roughly constant with increasing numbers of biosamples and does not vary with the complexity of component barcodes (Extended Data Fig. 2f), archetypal DHS elements must therefore encode regulatory information with extremely high linear density.

## Regulatory annotation of human genes

**Fig. 3:**
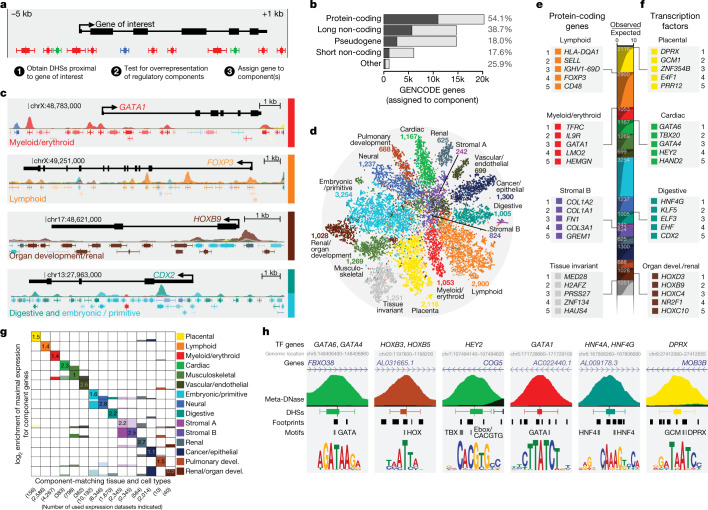
Regulatory annotation of human genes. **a**, Over-representation of DHS components in gene bodies and immediate flanks (maximum 5 kb upstream and 1 kb downstream). **b**, Percentage of genes annotated with DHS components (GENCODE gene categories). **c**, Regulatory annotation of *GATA1*, *FOXP3*, *HOXB9* and *CDX2* genes. **d**, Two-dimensional *t*-distributed stochastic neighbour embedding (*t*-SNE) projection of DHS component enrichment patterns across genes, coloured by dominant significant component (number of genes per component indicated). **e**, **f**, Summarized view of number of genes per component. Top five results for all protein-coding genes (**e**) and TF genes subset (**f**), for selected components. **g**, Correspondence between regulatory annotation and RNA expression shown using relative transcriptional activity across a panel of component-matched tissues and cell types (log_2_ observed/expected ratios). **h**, Putative TF-dependent regulatory elements defined by DHSs exclusively sharing regulatory components with genes encoding a given TF that also contain an occupied (footprinted) cognate TF motif.

**Extended Data Fig. 6:**
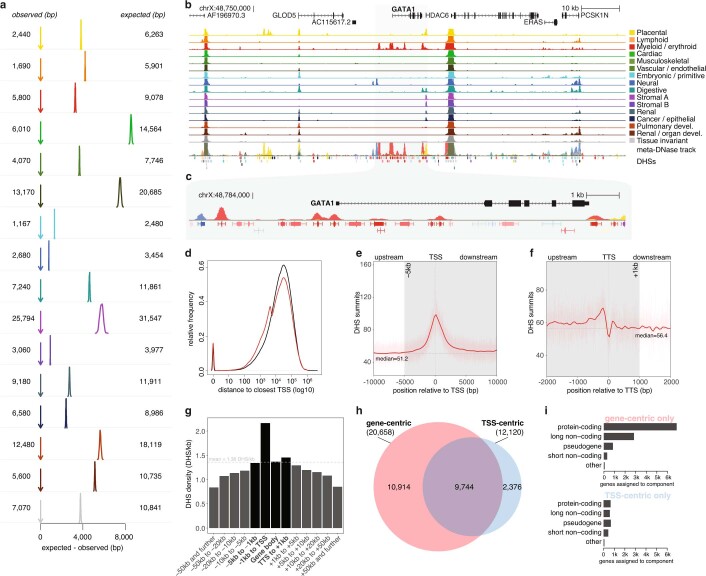
Clustering of same-component DHSs near genes. **a**, Component-specific genomic clustering of DHSs, as shown by the median distance between same-component DHSs as compared to the median distance after random permutation of DHS-component labels. **b**, Regulatory landscape +/− 50kb around the *GATA1* gene, indicating GENCODE gene annotations, meta-DNase tracks for individual DHS components (Methods), and a meta-DNase overlay track. **c**, Detailed view, restricted to the *GATA1* regulatory landscape, including its delineated and annotated DHSs. Collectively, this landscape shows a statistical over-representation of DHSs associated with the myeloid/erythroid (red) component. **d**, Density plot of DHS distance to the closest TSS for all Index DHSs (black line) and the subset (65%) of DHSs considered for the purpose of annotating genes using DHS components. **e**, **f**, Alignment plots showing DHS summit density across the transcription start sites (TSSs, **e**) and transcription termination sites (TTSs, **f**) of annotated genes. Shaded areas indicate regions included for the purpose of annotating genes using DHS components. **g**, DHS density expressed in terms of number of DHSs per kilobase, indicating a general enrichment of DHSs in and immediately surrounding genes. **h**, Venn diagram showing the overlap between regulatory annotations based on the gene-centric approach described in this work and a TSS-centric approach (+/−5kb). The gene-centric approach captures the vast majority of genes annotated using the TSS-centric approach, while adding an additional approximately 11,000 genes. **i**, Type of genes annotated using a gene-centric versus TSS-centric approach, showing the former yielding larger fractions of protein-coding and long non-coding genes.

The function of many genes is closely connected to their regulated expression across cells and tissues, and hence to the activity spectra of their cognate regulatory elements. We found that DHSs with similar component annotations were highly clustered along the genome (Extended Data Fig. 6a, b, Methods), particularly over gene bodies and their immediate flanking regions (Extended Data Fig. 6c), which collectively capture 65% of all DHSs (Extended Data Fig. 6d–g). We thus reasoned that integration of the components of DHSs overlying a given gene could be used to annotate its likely functional compartment(s). The existence of coordinately regulated DHSs in gene bodies cannot be ascribed to transcriptional activity per se, which produces only very minor changes in the general DNase I sensitivity baseline. Quantification of the enrichment of congruently annotated DHSs around 56,832 GENCODE genes (protein-coding and non-coding) genome-wide revealed 20,658 genes (5% FDR) with significant clustering along the genome of DHSs that belonged to the same component (Fig. 3a–d, Supplementary Table 2). Notably, the gene body-centric approach to annotation captured 70% more genes, and more genes that are likely to be biologically significant, than a TSS-centric approach (Extended Data Fig. 6h, i). Only a subset of gene body DHSs contributed to component assignments (median, 38%; IQR, 26–54%), consistent with the fact that many distal regulatory elements localize within the bodies of genes other than the ones that they regulate.

**Extended Data Fig. 7:**
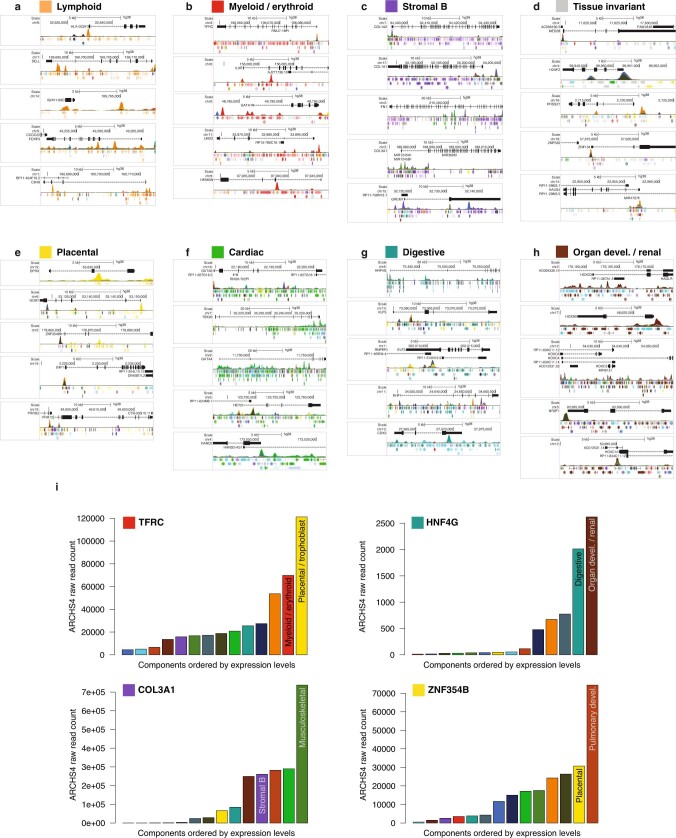
Top labelled genes for selected components. **a**–**d**, Top-scoring protein-coding genes per DHS component reflect their functional roles, as shown for lymphoid (**a**), myeloid / erythroid (**b**), stromal (**c**) and tissue-invariant (**d**) components. **e**–**h**, Top-scoring transcription factor (TF) genes per DHS component reflect their functional roles, as shown for placental (**e**), cardiac (**f**), digestive (**g**) and organ developmental/renal (**h**) components. Full gene regulatory landscapes used for labelling are shown, with GENCODE gene annotations, meta-DNase overlay track, and DHSs. **i**, Examples of component-annotated genes with discordant expression patterns. Coloured squares next to gene names indicate relevant components, in this particular case discordant with cell and tissue types with maximal expression.

Of 20,291 GENCODE protein-coding genes, more than half (54.1%) could be assigned a regulatory component based on their overlying DHSs (Fig. 3b). To determine whether these assignments were concordant with other functional annotations, we assessed (i) whether the genes most confidently annotated by a given DHS component reflected their known function(s), and (ii) whether genes annotated with a particular component are maximally expressed in cell types that match or are closely related to those components. The top genes annotated by the lymphoid component are all involved in immune response and disease (Fig. 3e, Extended Data Fig. 7a). Similar relationships were observed for other categories of gene (Fig. 3e, Extended Data Fig. 7b–d), including those annotated by the myeloid/erythroid component (erythropoiesis or haematopoietic stem cell genes), a stromal component (collagen genes and fibronectin), and the tissue-invariant component (housekeeping genes). This phenomenon was particularly notable for TF-encoding genes^30^ such as lineage-specifying master regulators of cardiac development (cardiac component; Fig. 3f, Extended Data Fig. 7f) or the development of other organ systems (Extended Data Fig. 7e–h).

To explore the concordance between DHS vocabulary annotations and gene expression across cell states, we investigated an independently generated compendium of more than 100,000 uniformly processed RNA sequencing (RNA-seq) datasets^31^. After matching DHS components with tissue-relevant expression datasets (Methods), we found strong correspondence between the vocabulary-based annotation of genes and the cell or tissue types in which they were maximally expressed (Fig. 3g). In many instances, DHS vocabulary annotation and gene expression offered different but complementary views of gene characteristics. For example, the transferrin receptor (TFRC) is responsible for cellular iron uptake and is required for erythropoiesis. *TFRC* RNA is most highly expressed in tissues from the placental component (Extended Data Fig. 7i), where TFRC is known to be involved in trophoblast membranes. From the perspective of regulation, however, *TFRC* is the most strongly associated gene in the myeloid/erythroid component (Fig. 3e, Extended Data Fig. 7b), in line with its core iron transport functionality. Analogously, the gene for HNF4G, a TF that is crucial for liver development, is the most strongly associated gene in the digestive component (Fig. 3e, Extended Data Fig. 7g); however, its RNA is expressed most highly in cells and tissues encompassed under the renal component (Extended Data Fig. 7i). DHS-centric annotations thus provide an orthogonal yet complementary view relative to expression data alone by providing a window into regulation. As DHSs greatly outnumber genes, DHS landscapes are in principle more information-rich than gene expression data alone.

## Annotating genes with unknown functions

**Extended Data Fig. 8:**
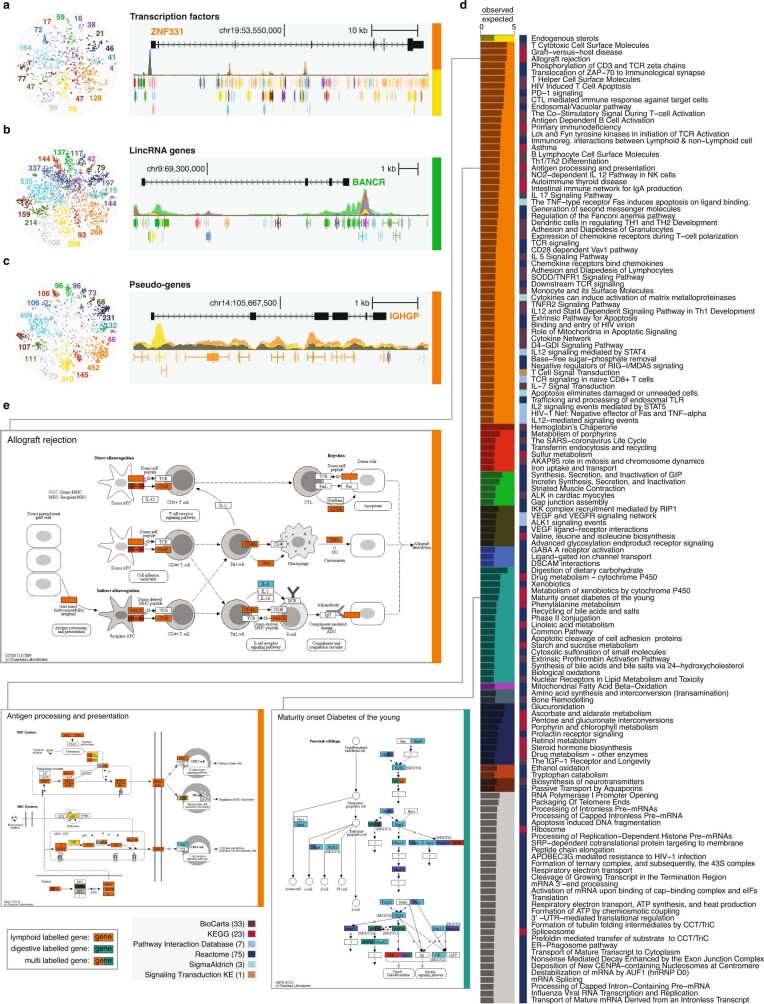
Annotation of genes with unknown function and pathways. **a**–**c**, Two-dimensional projection coordinates generated using *t*-SNE on all genes significantly associated with a DHS component and shown selectively for subsets of gene categories, namely transcription factors (TFs; diamonds: ZNF TF genes) (**a**), lincRNA genes (**b**) and pseudo-genes (**c**). Indicated are the number of labelled genes in each combination of gene category and DHS component. Examples of labelled genes are shown as follows. **a**, Regulatory landscape of *ZNF331*; a poorly annotated zinc-finger (ZNF) TF gene (lymphoid and placental components). **b**, Regulatory landscape of *BANCR*; a long intergenic non-coding RNA (lincRNA) gene, recently associated with cardiomyocyte migration. **c**, Regulatory landscape of the pseudo-gene *IGHGP* (lymphoid component). **d**, DHS component labelling of MSigDB canonical pathways, through the regulatory landscapes of constituent genes. Shown are pathways with a significant association (5% FDR) and an observed/expected ratio of at least 2. The most strongly associated components are indicated for each pathway, with their source databases. **e**, Examples of three component-associated pathways from the KEGG database, with genes coloured according to their majority component.

Despite intensive study, the function of many human genes remains obscure, particularly for those that are expressed at low levels or that have highly cell-selective expression patterns—for example, zinc-finger (ZNF) TFs^30,32^ or long non-coding RNA genes^33^. Nearly half of ZNF TFs (43.7%) could be annotated with a DHS component (Extended Data Fig. 8a), indicating their likely biological sphere of activity. Among long non-coding RNA genes, 38.7% could be mapped to DHS components (Extended Data Fig. 8b), as could 18% of pseudogenes^34^ (Extended Data Fig. 8c), which might reflect remnants of regulatory states that existed before ancient gene duplications. Beyond genes, we reasoned that entire pathways could be annotated using the DHS landscapes of their constituent genes (Extended Data Fig. 8d). For instance, the Kyoto Encyclopedia of Genes and Genomes (KEGG)^35^ pathway ‘allograft rejection’ (a paradigmatic immune response) is strongly enriched for the lymphoid component (Extended Data Fig. 8e), consistent with the concept that genes involved in similar biological processes should share similar patterns of regulatory element activity.

## Connecting DHS actuation to specific TFs

We reasoned that the coalescence of congruently annotated DHSs and genes, plus the availability of high-quality motif databases and newly available DNase I footprinting data^25^, could enable the systematic discovery of regulatory regions for which actuation patterns were likely to be driven, at least in part, by particular TFs. We identified 454 TFs with known sequence recognition motifs for which the encoding genes were annotated by a DHS component. We next identified 189,318 DHSs genome-wide (per TF median 149, IQR 47–477 DHSs) that (i) were exclusively annotated by a component matching that of the TF gene, and (ii) showed occupancy of the cognate motif by footprinting^25^ in a component-matched biosample (Fig. 3h). Such DHSs are likely to be highly functionally dependent on their associated TF, and should provide a rich substrate for experimental manipulations to investigate connections between TFs and regulatory functions.

## Annotating genetic association signals

**Fig. 4:**
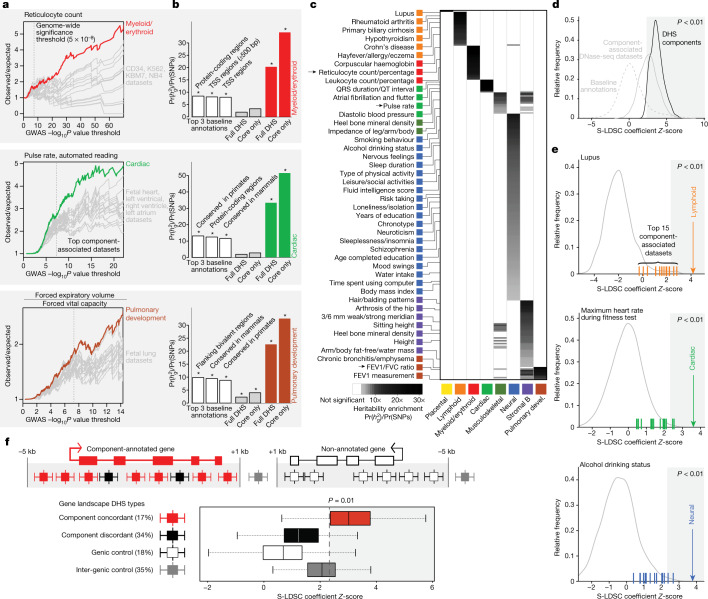
DHS components illuminate genetic associations and heritability. **a**, Association of DHSs with GWAS traits by component, shown as enrichment ratios for increasingly stringent subsets of variants (canonical genome-wide significance threshold of 5 × 10^−8^ indicated). Grey, enrichments for top 15 component-associated biosamples. **b**, Stratified LD-score regression (S-LDSC) for traits shown in **a** associates GWAS variants and DHS components. Heritability enrichment for the top three most enriched baseline annotations (white); the full DHS index (grey); and trait-relevant DHS components (red). *Statistically significant enrichment (one-sided test, 1% FDR). **c**, Enrichment of DHS component (*x*-axis) heritability across 261 GWAS traits (*y*-axis). Greyscale indicates heritability enrichment levels for statistically significant associations (one-sided tests, 1% FDR). Right, sampling of labels of enriched traits for each component. Arrows, traits from **a** and **b**. **d**, Distribution of S-LDSC coefficient *z*-scores across 261 GWAS traits, shown for all baseline annotations (dashed grey line), top 15 DHS component-associated biosamples (solid grey line) and DHS components (black line). **e**, S-LDSC coefficient *z*-scores for selected traits (lupus, *q* = 0.002; maximum heart rate during fitness, *q* = 0.016; alcohol drinking status, *q* = 0.009), shown for all biosamples (grey lines), top 15 component-associated biosamples (coloured ticks) and DHS components (coloured arrows). **f**, Stronger heritability contribution of component-concordant DHSs shown by stratifying S-LDSC *z*-scores by DHS types. Boxes, medians and IQRs (25–75%); whiskers, 1.5 × IQRs; *n* = 261 GWAS traits. Grey areas in **d**–**f** indicate S-LDSC *z*-scores (S-LDSC coefficients, normalized using estimated standard errors) with *P* < 0.01; FDR-corrected *q*-values shown for traits in **e**.

**Extended Data Fig. 9:**
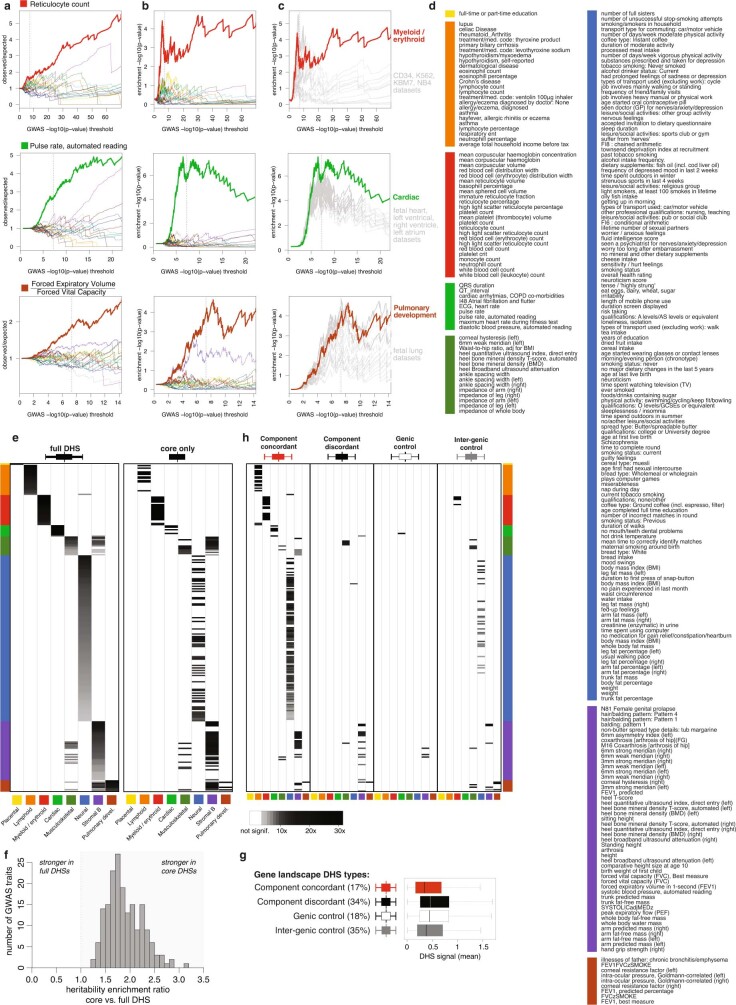
GWAS trait associations of DHS components. **a**–**c**, Quantitative association of component-DHSs with GWAS traits reticulocyte count, pulse rate, and FEV1/FVC ratio. Canonical genome-wide significance threshold indicated (5 × 10^−8^). **a**, Enrichment ratios for increasingly stringent subsets of variants, per DHS component. **b**, Nominal enrichment −log_10_(*P* value) of a one-sided binomial test for each DHS component. **c**, Nominal enrichment -log_10_(p-value) of a one-sided binomial test for the strongest DHS component only, along with its strongest associated biosamples. **d**, GWAS traits associated with component-annotated index DHSs. **e**, Greyscale values, heritability enrichment levels for statistically significant (FDR 1%) traits based on the full delineated width of index DHSs (left) and restricted to index DHS ‘core’ regions (right). Row labelled as per **d**. **f**, Ratio of heritability enrichment values for ‘core’ versus ‘full size’ DHSs. **g**, DHS confidence scores (mean signal) stratified according to gene landscape DHS types. Boxes denote medians and interquartile ranges (IQRs, 25–75%), whiskers represent 1.5 x IQRs, *n* = 261 GWAS traits. **h**, Heritability enrichments stratified according to gene landscape DHS types. Greyscale indicates heritability enrichment levels for statistically significant associations (1% FDR).

We next investigated whether DHS annotations could expand insights into the role(s) of genetic variation in regulatory DNA, and thus provide a more meaningful framework for interpreting the pathophysiological basis of disease and trait associations. A rank-based analysis of disease or trait against DHS component associations (explicitly controlling for large scale linkage disequilibrium (LD) structure; Methods) revealed increasingly strong component-specific enrichments of association signals across diverse traits (Fig. 4a, Extended Data Fig. 9a, b). In many cases these enrichments exceeded those obtained by considering only DHSs detected in biosamples most closely related to the relevant DHS component (for example, lymphoid cell biosamples versus lymphoid component; Fig. 4a, Extended Data Figs. 4a, 9c).

Quantifying the extent to which DHS annotations captured SNP-based trait heritability^36^ (*h*_g_^2^) (Fig. 4b) revealed a strong increase in heritability enrichment for trait-relevant DHS components (Fig. 4b, coloured bars) relative to all index DHSs (Fig. 4b, grey bars) or to a large panel of 85 baseline annotations (Fig. 4b, white bars; top three annotations shown). Heritability was markedly enriched specifically within DHS ‘core’ regions, providing orthogonal evidence for the delineation and importance of this subcompartment (Fig. 4b).

To generalize these observations, we compiled more than 1,300 traits with SNP-based heritability of at least 1% from the UK Biobank project^37^ and from curated published data^38^. Of these, 261 diseases and traits showed highly significant component-specific enrichment in heritability, particularly for pathophysiologically relevant DHS components (Fig. 4c, Extended Data Fig. 9d; 1% FDR). Restricting DHS delineations to ‘core’ regions again yielded significantly greater enrichment compared to full DHSs (Extended Data Fig. 9e, f).

To remove potentially confounding contributions from multiple genomic annotations that overlap the same SNP (for example, a DHS that overlaps a coding region of a gene annotated with a particular DHS component), we quantified the statistical significance of DHS component heritability contributions while controlling for the contribution of all other annotations (Methods). For virtually all reported traits, DHS component annotations significantly (*P* < 0.01) captured SNP-based trait heritability (Fig. 4d, black line).

We next performed cell type-specific heritability analyses^39^ to quantify the concentration of trait-associated genetic signals in DHSs annotated by specific DHS components, relative to the full repertoire of DHSs mapped in disease- or trait-relevant cell types (Methods). Component-annotated DHSs produced significant improvements in capturing trait heritability compared to individual biosample maps (*P* < 2.2 × 10^−16^; Fig. 4d, grey solid line). At the level of specific traits, in 68 out of 261 cases (26%), DHS component annotations captured trait heritability better than individual DNase-seq datasets (Fig. 4e). We conclude that the current index of highly resolved consensus DHSs markedly sharpens disease and trait association and heritability signals.

## Genetic signals span gene body DHSs

The observed clustering of concordantly regulated DHSs along gene bodies (Fig. 3) led us to speculate that such DHSs were more likely than other DHSs to contain relevant genetic signals. To test this idea, we quantified trait heritability separately for component-concordant DHSs (17% of DHSs) and component-discordant DHSs (34%) within gene bodies (Fig. 4f). Concordant DHSs strongly contributed to SNP-based trait heritability relative to DHSs that were found in the same genes but with component annotations discordant with the annotations of the underlying gene, despite having lower average DNase-seq signal levels (Extended Data Fig. 9g) and more specialized utilization patterns (occurring in an average of 15 versus 25 biosamples). DHSs that were proximal to genes not labelled by any DHS component showed the weakest heritability contributions, and intergenic DHSs contributed only modestly (Fig. 4f, Extended Data Fig. 9h). Rather than being confined to a small number of distal elements or promoters, it thus appears that genetic association signals are concentrated within congruently regulated sets of DHSs that decorate entire gene bodies.

## Discussion

Here we have presented the most comprehensive and precise map of human DHSs, and a common coordinate system and vocabulary for regulatory DNA, creating a framework for global analyses of tissue-specific gene regulation and its intersection with human disease trait genetics. Regulation across cell types and states is a cardinal property of DHSs that is now captured in DHS components, greatly expanding the analytical horizon beyond cell type-agnostic annotations such as chromatin states^7,40^. Common reference coordinates should additionally facilitate comparisons between large experimental datasets, and between human and mouse DHSs, which can now be directly linked in a manner that is robust to future mouse assemblies^17^.

Given the scale of the data, it is natural to ask how complete and stable our current maps are. New biosamples will add new DHSs and annotate existing elements with ever higher precision. Adding 733 biosamples of equivalent biological breadth would increase the number of consensus DHSs by an average of 27%, with rapidly diminishing returns after that. From the current 21.55% it also is reasonable to predict that no more than 28% of the extant human reference sequence encodes *cis*-regulatory modalities that give rise to DHSs.

It should now be possible to triangulate the genetics-to-gene-regulation interface along three axes: (i) a genomic position axis, which is now finely resolved to consensus DHS summits (centroids); (ii) a cell/tissue-state axis now captured in DHS components; and (iii) a gene context axis that reflects the coherent co-localization of similarly regulated DHSs over gene bodies. The convergence of GWAS variants in coordinately regulated gene body DHSs suggests a fundamental feature of the genetic architecture of disease that has heretofore, to our knowledge, escaped notice. This finding resulted from combining the sharpened disease association and heritability signals enabled by high-precision annotation of regulatory DNA with the new ability to annotate the biological spectrum of each element, neither of which would have been possible without the large advances in biological scale and methodologies reported here. The fact that genetic association and heritability signals are concentrated across congruently regulated sets of DHSs that decorate entire gene bodies has important theoretical and practical implications for understanding both the genetic architecture of disease and the problem of connecting genetic signals with their target genes, which is critical for therapeutic translation.

**Extended Data Fig. 10:**
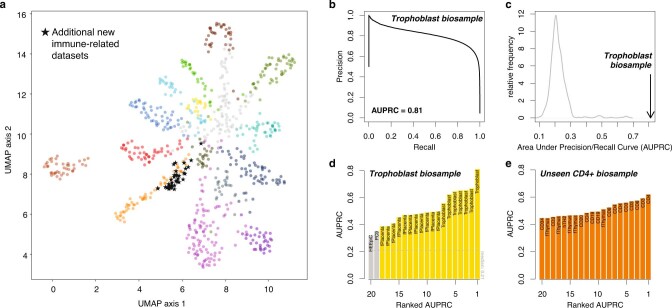
Extendability of the DHS annotation framework. **a**, Two-dimensional UMAP projection of 733 biosamples by way of their index DHS utilization, coloured by their strongest representative NMF component. Stars indicate the embeddings of 38 previously unseen immune-related DNase-seq peak call datasets. **b**, Area under precision recall curve (AUPRC) values for predicting per-biosample DHSs from DNase-seq signal alone, shown for a trophoblast biosample. **c**, AUPRC values for the matching trophoblast versus all other 732 biosamples. **d**, Top 20 biosamples matching the aforementioned trophoblast biosample in terms of AUPRC values. **e**, Top 20 biosamples (out of 733) matching an unseen CD4+ biosample in terms of AUPRC values.

More broadly, the framework we report represents a transition from an exploratory era focused on the discovery of novel elements, to a map-centric era with a focus on the detection of previously annotated elements within specific biological contexts (Extended Data Fig. 10a). The index framework may also obviate the need for peak calling (Extended Data Fig. 10b–e), and should prove particularly valuable for anchoring single-cell studies^41^, which are presently at least 1,000-fold too sparse for robust delineation of regulatory DNA within individual cells.

## Methods

### Generation of DNase I hypersensitivity maps

DNase I assays were generally performed according to a protocol detailed previously^42^. This protocol involves treatment of intact nuclei with the small enzyme DNase I which is able to penetrate the nuclear pore and cleave exposed DNA. Small (<1 kb) fragments are isolated from lysed nuclei following DNase I treatment, linkers are added, and the resulting library is sequenced. Because tissue and cell culture, isolation, and handling protocols differ for different biosamples, these are indexed in Supplementary Table 1. Additional information on the procurement of biosample material and DNase-seq biosample selection and data processing is available in the Supplementary Methods.

### Index of consensus human DHSs

DHSs were detected in individual biosample datasets and integrated across all 733 datasets to yield a set of 3.59 million consensus DHS delineations. These elements were subsequently annotated with estimates of their centre-of-mass, positional stability across datasets and confidence scores. A detailed explanation of this procedure is provided in the Supplementary Methods.

#### Overlap of the DHS index with genomic annotations

To assess the overlap of our DHS consensus elements with repetitive elements (Extended Data Fig. 2b), we obtained RepeatMasker^43^ annotations downloaded from the University of California Santa Cruz (UCSC) Table Browser^44^, and considered the various repeat classes and (sub)families as provided. To perform analogous analyses for human gene annotations (Extended Data Fig. 2c), we obtained GENCODE^45^ v.28 Basic annotations. We defined exons as specified in the GENCODE annotation, promoters as the TSS of genes ±1 kb, and introns as the rest of the gene body. Intergenic regions were defined as those not covered by gene bodies or defined promoters. We assigned index DHSs to these annotations requiring at least a 1 bp overlap, choosing the annotation with the largest overlap in case of multiple overlapping annotations.

TOPMed within-human sequence variation data were obtained from the Bravo website (https://bravo.sph.umich.edu/freeze5/hg38/download, Freeze 5, hg38, VCF format). We converted 495.6 million single-base substitutions to nucleotide diversity scores (*π*), with a score of zero implied for every genomic base position with no variants. Per base, phyloP^46^ sequence conservation scores were downloaded as-is (http://hgdownload.cse.ucsc.edu/goldenpath/hg38/phyloP100way/). Within-human sequence variation data (*π* × 10^4^) and phyloP conservation scores were aligned relative to DHS centroids using 20-bp non-overlapping windows tiled across a 1-kb region centred on each centroid (Extended Data Fig. 2g). For each window offset relative to the DHS centroid, genome-wide per-base scores were subsetted using bedops^47^ and averaged with GNU datamash.

#### Saturation and extendability of DHS index

For random subsamples of sizes ranging from 1 to 733 biosamples, we estimated the mean number of novel DHSs added by a new dataset as a function of total number of datasets sampled (Extended Data Fig. 2h). To extrapolate these estimates to future biosample sets, we fitted a log-log model to the data. From the saturation analysis, we expect the overwhelming majority of DHSs identified in any new dataset to be represented already in the index, to which they will contribute additional confidence and precision. Incremental datasets can be added to the index by re-delineating DHSs using the original per-dataset DHS calls permanently recorded at the ENCODE DCC (Supplementary Table 1).

### Construction of a DHS vocabulary

We used NMF^28,29^ for the decomposition of a binary matrix consisting of the presence or absence calls of *m* DHSs across *n* DNase-seq datasets into a smaller set of *k* components. As with other dimensionality reduction methods, NMF does not guarantee a total recapitulation of the original data; instead we chose to allow information loss in exchange for a more interpretable result. Therefore, we considered using a much smaller number of *k* components than the lower of the two dimensions of our input matrix (733 DNase-seq datasets). To keep the reconstruction error in check, we used an objective function that is minimized subject to the Frobenius norm (Extended Data Fig. 3a). NMF typically uses a random initialization step, leading to unstable results. To alleviate this, we performed the initialization step using singular value decomposition (SVD)^48,49^, leading to consistent results while maintaining a performance that is on par with randomly initialized instances. A more detailed rationale for the component-wise description of DHSs, as well as details on the implementation and execution of the decomposition, is provided in the Supplementary Methods.

#### Labelling of NMF components and DHSs

To aid interpretation of the 16 NMF-derived components, we used two orthogonal approaches to assign labels to components, based on (i) biosample properties and (ii) DHS sequence features.

First, for each component we selected the top biosamples based on component-specific NMF loadings present in their datasets (Extended Data Fig. 4a). These maximal NMF loadings across datasets were generally strong across components (Extended Data Fig. 4b). In general, a clear pattern emerged of shared properties of biosamples most strongly associated with specific components. To formalize this, we performed one-sided Mann–Whitney *U* tests to assess whether NMF loadings for biosamples sharing certain metadata categories (Supplementary Table 1) are greater than those for biosamples not in the given metadata category (Extended Data Fig. 4c). In particular, we assessed metadata categories corresponding to human organ systems and the cancer status of biosamples. *P* values were corrected for multiple hypothesis testing using the Bonferroni correction method. A post hoc analysis of biosample-to-component assignment for values of *k* < 16 provided insight into the genesis of our *k* = 16 component model, showing junctures after which separate cell type lineages are captured by distinct components (Extended Data Fig. 4d).

Second, for each component we obtained DHSs with maximal NMF loadings for that component, and subsequently performed enrichment analyses for TF binding site motifs (Extended Data Fig. 4e). We used a wide array of TF motifs and used FIMO^50^ (match threshold *P* < 10^−5^), to search for motif instances in the human genome. We tested the association of motif occurrences with specific NMF components using Fisher’s exact test. We used clusters of similar motifs (http://www.mauranolab.org/CATO/weblogos/main.html) for the purpose of summarization and visualization. The results show strong enrichments for component-specific motifs, suggesting preferential binding of component-relevant transcription factors (Extended Data Fig. 4f).

The strong associations of 1) biosample properties and 2) TF binding site occurrences with specific components enabled us to label each NMF component, resulting in a DHS vocabulary (Fig. 2d), further detailed in the Supplementary Note. For downstream analyses, we labelled each DHS with its strongest NMF component (Fig. 2e, bottom).

#### Robustness of component interpretation

To test the effect of inducing additional sparsity in the NMF model, we systematically increased the L1 penalization setting while tracking F1 scores and the fraction of non-zero parameters used in the model (Extended Data Fig. 5a–c). The top 15 component-contributing biosamples per component remain mostly consistent with Fig. 2e and Extended Data Fig. 4a without L1 penalization, indicating that enforcing additional sparsity does not impact the interpretation of model components.

To test the effect of possible over/under-representation of certain cell types, we removed 44 (40%) haematopoietic biosamples, consisting of the highest quality datasets representative of unique cellular conditions (Supplementary Table 1). After building a new NMF model, we observe that although the remaining (lower quality) haematopoietic biosamples are now captured by a single component instead of two, the interpretation of the remaining non-haematopoietic components does not change (Extended Data Fig. 5d).

### Regulatory annotation of human genes

#### Per-component genomic distribution of DHSs

We compared the average distance between same-component DHSs against empirical distributions based on random assignment of component labels to DHSs and sampling the same number of DHSs 1,000 times (Extended Data Fig. 6a).

#### Per-component meta-DNase tracks

To illustrate the regional diversity of DHS component data, we generated meta-DNase tracks representing each of the 16 DHS components (Extended Data Fig. 6b) by averaging genome-wide DNase-seq signal profiles of the top 15 biosamples most strongly associated with each component (Extended Data Fig. 4a). For visual conciseness, we provide aggregate tracks that overlay the meta-DNase tracks of all DHS components (for example, Fig. 3c, Extended Data Figs. 6b, c, 7a–h, 8a–c).

#### Definition of regulatory landscapes

We defined the regulatory landscape of a gene as the set of DHSs within the gene body, plus DHSs in flanking regions of maximally 5 kb upstream and maximally 1 kb downstream of the gene body, or up until halfway through to the gene upstream, whichever value is smaller (Fig. 3a, Extended Data Fig. 6e–g). This captures approximately 65% of all DHSs (Extended Data Fig. 6d) and prevents flanking region DHSs from being routinely assigned to the regulatory landscapes of multiple genes, alleviating mixing of regulatory signals.

#### Association of genes with DHS components

We tested the association of all 56,832 annotated GENCODE genes (Fig. 3b) with each DHS component separately. Under the null hypothesis that DHS components are randomly distributed across gene regulatory landscapes, we used the binomial distribution to test whether the proportion of DHSs annotated with a given component is higher among DHSs within a particular gene regulatory landscape than outside. We controlled the FDR at 5% by calculating *q* values^51^ across the total of all genes and components. Further details are provided in the Supplementary Methods. To study the differences between a gene-centric and TSS-centric approach, we calculated component associations for 10-kb regions centred around the TSS (that is, TSS ± 5 kb) and assessed the number and type of genes annotated (Extended Data Fig. 6h, i).

#### Annotations for GENCODE genes and pseudo-gene types

GENCODE v.28 (Basic) annotations were used for all analyses. For the purpose of labelling and visualizing genes, for each gene we used its longest transcript as its representative region. Pseudo-gene annotations were obtained from psiCube^52^, http://pseudogene.org/psicube/data/gencode.v10.pgene.parents.txt.

#### Visualization of gene regulatory annotations

We used *t*-SNE to visualize the enrichment ratios of gene regulatory landscapes for DHS components (Fig. 3d, Extended Data Fig. 8a–c). Each dot shown represents a gene found to be significantly associated with one or more DHS components, and the union of these are the genes used to calculate the 2D embedding. The R (http://www.r-project.org) implementation as provided in the Rtsne package was used, with default parameters. Genes are coloured according to their (most strongly enriched) significant DHS component.

#### Construction and use of gene expression compendium dataset

We used the full human ARCHS4 dataset (downloaded 26 June 2018)^31^ and selected relevant tissue and cell types for each DHS vocabulary component (Supplementary Methods). This resulted in a total of 33,733 unique gene expression datasets, with expression information for 35,238 genes. For each gene, we obtained the 95th percentile value across datasets selected for each DHS component as the representative value in that component, to not be led by outliers in the data, while still being sensitive for cell type selective expression levels. For each DHS component, we calculated average expression levels across genes labelled with that component (observed), as well as across all component-labelled genes (expected). Resulting values are reported as log_2_-transformed enrichment ratios (Fig. 3g).

#### Annotation and visualization of pathway labellings

A curated set of canonical pathways was obtained from the MSigDB Collections (http://software.broadinstitute.org/gsea/msigdb/genesets.jsp?collection=CP). Pathway enrichment analyses (Extended Data Fig. 8d, e) were performed analogously to gene enrichment analyses, by pooling DHSs in neighbourhoods of all pathway-associated genes. We used the KEGG^35^ REST API (https://www.kegg.jp/kegg/rest/keggapi.html) to download and graphically annotate KEGG pathway representations.

#### Prioritization of TF-associated DHSs

We obtained DHSs with loadings for a single component only. For each component-labelled TF gene with a known sequence binding motif, we obtained the subset of DHSs that (i) are annotated with the same component as the TF, (ii) contain a TF-matching motif, and (iii) are footprinted in a biosample associated with the same component^25^ (Fig. 3h). Although the above analysis identified a small minority of DHSs owing to stringent filtering, motifs with variable information content, and the smaller range of biosamples for which footprinting data are available, this approach could be recapitulated with less extreme parameters to identify larger sets of DHSs at reasonable confidence.

### Genetic variation analyses

#### GWAS traits and summary statistics

We obtained GWAS summary statistics data from the UK Biobank project as processed by the Neale lab (http://www.nealelab.is/uk-biobank/). In addition, we obtained GWAS summary statistics calculated using BOLT-LMM v2.3^53^, as used in recent work^38^.

#### Estimates of SNP-based heritability

GWAS traits were curated by removing those with a narrow-sense SNP-based heritability^54^ (*h*_g_^2^) of less than 1%. Although ideally we would quantify heritability by considering the true causal effects of variants, in reality we do not observe these. Instead, we are limited to GWAS summary statistics, which essentially describe the marginal trait-correlation for each variant, consisting of both causal effects and effects due to LD, plus statistical noise. Recently proposed methods such as LD score regression (LDSC)^55^ are able to estimate heritability while explicitly considering the underlying LD structure. For continuous traits, in case both raw and inverse-ranked normalized (irnt) versions were available, we retained the latter only. This yielded a total of 1,316 traits for subsequent analyses with an *h*_g_^2^ of at least 1%.

#### Quantitative trait associations

For quantitative trait-versus-component analyses (Fig. 4a, Extended Data Fig. 9a–c), we assessed the correspondence between trait association strength (GWAS variant association *P* value) and the component annotations of variant-containing DHSs, for increasingly stringent subsets of GWAS variants. Enrichment *P* values were calculated using a binomial distribution, as done previously^6^. We explicitly control for large scale LD structure, using a form of LD clumping^56^, by selecting a single variant-containing DHS for each of 1,708 approximately independent LD blocks^57^. Namely, for each LD block, the variant with the lowest GWAS association *P* value that overlaps a DHS was selected for subsequent analysis. In case multiple such variant-containing DHSs existed, we gave preference to the DHS with the highest confidence score (mean signal) in our DHS index.

#### Stratified LD-score regression

To estimate *h*_g_^2^ with maximal statistical power, we used LD score regression (LDSC)^55^ to explicitly take into account LD structure. In particular, we used a stratified version of LDSC (S-LDSC)^36^ to partition heritability estimates according to pre-defined sets of genome-wide annotations (Fig. 4b, c, Extended Data Fig. 9d, e), consisting of our annotated DHSs in addition to a wide range of 85 genome-wide functional ‘baseline’ annotations (baseline-LD model v.2.1). The v.2.1 baseline set consists of a total of 86 genome-wide annotations, building upon the 76 annotations used in the v.2.0 set and several additional annotations^58^. These ‘baseline’ annotations encode whether SNPs fall inside protein-coding or non-coding regions, regions with increased levels of evolutionary conservation, regions predicted or confirmed to have enhancer activity, and so on. Their breadth provides a robust^36^ baseline model along which to test trait heritability contributions of our DHS components. We express the heritability enrichment of an annotation as the ratio of its proportion of per-trait *h*_g_^2^ and the proportion of SNPs covered by the annotation (Fig. 4b).

Variants included in the analysis are those registered in HapMap3, with a minimal minor allele frequency (MAF) of 5%, and excluding the human major histocompatibility complex (MHC) locus. Baseline LD scores were computed from 1000 Genomes Phase 3 data from European ancestry populations and corresponding allele frequencies (as used previously^58^ and available from the LDSC reference downloads page, along with the baselineLD annotation set: https://data.broadinstitute.org/alkesgroup/LDSCORE/).

#### Heritability enrichments for DHS vocabulary components

We applied S-LDSC to our DHS vocabulary components as follows. In brief, each DHS was assigned to its majority DHS component and (when possible) assigned to overlapping variants. For the resulting vocabulary-based annotations, LD scores were calculated. We then performed S-LDSC separately for each of the selected 1,316 traits, relative to these vocabulary-based annotations and the baselineLD model described above. For each trait versus annotation combination, we obtained estimates of its heritability enrichment^36^, expressed as the ratio of its proportion of *h*_g_^2^ and the proportion of SNPs covered by the annotation (Fig. 4b, c). We considered heritability enrichments statistically significant at an estimated FDR of less than 5% calculated across all considered traits and DHS components. This is more stringent than the commonly used per-trait correction for multiple hypothesis testing.

#### Unique per-annotation contributions to SNP-based heritability

Estimates of heritability enrichment can be confounded by contributions of multiple (overlapping) genomic annotations included in S-LDSC models. To quantify unique per-annotation contributions to heritability, we obtained the average per-SNP increase in heritability ascribed to that component, after controlling for all other annotations in the model (baseline annotations and DHS components)^36^. From the reported coefficients and their standard errors, we derived *z*-scores, one-sided *P* values and FDR-corrected *q* values for each trait-versus-component combination (Fig. 4d, e). For the heritability analysis in component concordant genic DHSs (Fig. 4f), we further stratified DHSs based on whether they are component concordant, component discordant, inside non-annotated genes (genic controls), or inter-genic. Figure 4f shows *z*-scores for the maximally enriched components identified in Fig. 4c.

To quantify the heritability contribution of per-dataset DHSs, we performed a variation on the standard S-LDSC procedure, as described previously^36^. Specifically, we built upon the baselineLD model by iteratively considering annotations derived from individual datasets only. These individual datasets were collected by selecting for each trait the 15 datasets most informative to each DHS component (Extended Data Fig. 4a). Annotations consist of DHSs observed in those datasets, as well as their complement, that is, the remainder of index DHSs. We report the contribution to heritability based on the former, expressed as *z*-scores (Fig. 4d, e).

### Extendability of the DHS vocabulary

#### Addition of novel unseen datasets

New datasets may be added to the current NMF model while retaining the same interpretation of components (Extended Data Fig. 10a). In brief, 0.1% FDR variable-width peak calls are obtained from new datasets of interest, mapped to DHS index elements using bedops^47^ and projected into the existing component space using standard NMF routines (see code for more details).

#### DHS index element identification without de novo peak identification

We used bedops^47^ to look up DNase-seq signal levels of a dataset of interest over index elements, to determine whether a given element is actuated in the dataset. Expressed as a classification problem, using the existing 0.1% FDR variable-width peak calls as the groundtruth set, we assess precision and recall of peak recovery. For all 733 biosamples we find area under precision recall curve (AUPRC) values ranging from 0.33 to 0.83 (median, 0.71; IQR, 0.64–0.75), with a trophoblast biosample (ENCODE DCC identifier ENCBS576QRR) shown as an example (Extended Data Fig. 10a). The large difference between AUPRC values of matched versus non-matched biosamples allows the identification of the original biosample (Extended Data Fig. 10b), while showing that biosamples with similar AUPRC ranks share the same biological characteristics (Extended Data Fig. 10c). This procedure can also be followed for unseen datasets (Extended Data Fig. 10d), in particular datasets that are less deeply profiled or would otherwise be too sparse to call peaks on de novo—such as single cell chromatin profiling data.

### Reporting summary

Further information on research design is available in the Nature Research Reporting Summary linked to this paper.

## Online content

Any methods, additional references, Nature Research reporting summaries, source data, extended data, supplementary information, acknowledgements, peer review information; details of author contributions and competing interests; and statements of data and code availability are available at 10.1038/s41586-020-2559-3.

## Supplementary information

Supplementary Methods This file contains Supplementary Methods, Supplementary Note and Supplementary References.

Reporting Summary

Supplementary Table Supplementary Table 1 | Biosample metadata spreadsheet. Spreadsheet with per-biosample metadata, including biosample taxonomy and identifiers, experimental and computational quality measures of resulting datasets, and hyperlinks to experimental protocols. This Table is also available as a Google Spreadsheet: https://docs.google.com/spreadsheets/d/1B9WKRwXUr8h_frEyh5Zt09oZbnejYIM_LciBcNrZ6Ew/edit?usp=sharing

Supplementary Table Supplementary Table 2 | Gene labeling spreadsheet. Spreadsheet with per-component gene labelings.

## Data Availability

All primary data are available from the ENCODE DCC portal. Biosample metadata are available in Supplementary Table 1 as well as in other formats via Zenodo (10.5281/zenodo.3838751). The set of more than 3.5 million DHS delineations is available in tab-separated format from the ENCODE DCC portal (https://www.encodeproject.org/annotations/ENCSR857UZV/) and via Zenodo (10.5281/zenodo.3838751). Data matrices describing the occurrence patterns of DHSs across biosamples are available in various formats via Zenodo (10.5281/zenodo.3838751). There are no restrictions on data availability and (re)use. We additionally provide a specialized data browser (https://index.altius.org/) and a trackhub for the UCSC Genome Browser (https://genome.ucsc.edu/cgi-bin/hgTracks?db=hg38&hubUrl=https://resources.altius.org/~meuleman/DHS_Index_tracks/hub.txt). BED files documenting the coordinates and annotations of DHSs with evidence of being bound by specific transcription factors are available via Zenodo (10.5281/zenodo.3838751), and top-scoring elements per TF can be explored in a browser (https://index.altius.org/?application=viewer&roiSet=TFassoc_Meuleman).

## Code availability

Code is available on Github for building the index of consensus DHSs (https://github.com/Altius/Index), for constructing the DHS vocabulary and the addition of novel biosamples (https://github.com/Altius/Vocabulary).
