# Supplementary material for: Index and biological spectrum of human DNase I hypersensitive sites
Source: Nature. 2020 Jul 29;584(7820):244–51. doi: 10.1038/s41586-020-2559-3 (PMC7422677; doi:10.1038/s41586-020-2559-3)
Supplement: Supplementary file 1 — This file contains Supplementary Methods, Supplementary Note and Supplementary References. [file 41586_2020_2559_MOESM1_ESM.pdf]

nature

https://doi.org/10.1038/s41586-020-2559-3

Supplementary information

Index and biological spectrum of human DNase I hypersensitive sites

In the format provided by the authors and unedited

Wouter Meuleman✉, Alexander Muratov, Eric Rynes, Jessica Halow, Kristen Lee, Daniel Bates, Morgan Diegel, Douglas Dunn, Fidencio Neri, Athanasios Teodosiadis, Alex Reynolds, Eric Haugen, Jemma Nelson, Audra Johnson, Mark Frerker, Michael Buckley, Richard Sandstrom, Jeff Vierstra, Rajinder Kaul & John Stamatoyannopoulos✉

Supplementary Methods

Primary data generation and processing

Biosample material procurement

The source of all cell line and primary biosample material is described (when available) in and linked to from **Supplementary Table 1** (Google Spreadsheet). Many primary tissues are no longer available as they constituted unique "terminal" study material. All relevant biosample material consisted of anonymized samples received from centers that followed IRB approved protocols. Only NIH approved hESC lines were used in studies. Approval by ESCROW for their use was received, where necessary. These studies were declared as non-human subject studies. One included cell line (SK-N-MC) appears on the ICLAC list (http://iclac.org/databases/cross-contaminations/), and we properly document this biosample as Ewing's sarcoma as opposed to a neuroepithelioma biosample.

DNase-seq sample selection and data processing

Because regulatory DNA accessibility exhibits a wide dynamic range (hundreds-fold) across elements, the full extent of the accessible DNA landscape of a given cell type only becomes evident with high quality, high signal-to-noise data. At the high end of the spectrum are promoter elements, which exhibit high accessibility but little cell type-selectivity. By contrast, cell selectivity is concentrated in moderate-to-lower accessibility distal elements, which are under-sampled in lower quality data.

For this reason, we considered exclusively high quality data with a SPOT score (https://www.encodeproject.org/data-standards/terms/#enrichment) of at least 0.3 for our analyses, yielding a total of 733 DNase-seq datasets. Collectively, these data assay a total of 438 cell and tissue states spanning all human organ systems. We defined the term "cell and tissue state" to encompass distinct cell types; distinct developmental time-points in case of fetal tissue data; different time-points in case of

differentiating cultured cells; and (for a small number of datasets) different treatment conditions (**Supplementary Table 1;** Google Spreadsheet).

Processing of DNase-seq FASTQ files was done using the ENCODE DCC DNase-HS pipeline paired-end, version 2 (ENCPL202DNS). Briefly, reads were trimmed of adapter match sequence and subsequently aligned to the human genome reference GRCh38/hg38 using BWA (version 0.7.12). Filtered, quality mapped, reads from all sequencing runs of a given library were merged. Collectively across datasets, we obtained 94,091,469,219 (94 billion) usable mapped DNase I cleavage fragments.

Further processing and QC evaluation was performed in accordance with the ENCODE DCC pipeline specification (https://www.encodeproject.org/data-standards/dnase-seq/). Standardized output from the DCC pipeline includes normalized read densities, which are a sum of 5' read end counts in a 150bp sliding window across the genome. These values are reported at 20bp steps across the genome. In order to facilitate integrative analyses across datasets, these values are normalized by dividing each value by the total uniquely mapping read count and scaling by 1 million. DNase-seq datasets are available from the ENCODE Consortium Data Portal (http://www.encodeproject.org), including raw non-normalized and non-windowed versions.

Construction of an Index of DNA accessibility

For any given DNase I Hypersensitive Site (DHS) shared across a number of datasets, the precise observed location and width of said hypersensitive site may vary slightly between datasets. For this reason, an integrative analysis across multiple datasets requires a form of alignment of sites. In essence, each DNase-seq peak call represents an imperfect guess of where the actual hypersensitive site is. We exploited this to obtain more high-resolution and precisely delineated regulatory regions by combining evidence across datasets to arrive at a consensus delineation. The code for this procedure is available (https://github.com/Altius/Index) and a brief description follows.

Detection of strong peaks in individual datasets

Starting from per-dataset data tracks (**Extended Data Fig. 1b-c, step 1**), we detected statistically strong (0.1% FDR) variable-width peaks across individual datasets using the Hotspot2 software (https://github.com/Altius/hotspot2) over uniquely mappable regions of the genome. On average, each dataset was called with 104,433 peaks, for a total of 76,549,656 (76.5M) peaks across all 733 DNase-seq datasets (**Extended Data Fig. 1b-c, step 2**). For each peak, we registered their start, end and estimated summit positions, as well as their signal intensities.

Definition of isolated accessibility events

We used the local clustering of peak summit positions across datasets to define isolated events of chromatin accessibility, by aggregating the 1bp summit locations of peaks across datasets (**Extended Data Fig. 1b-c, step 3**). For computational efficiency, we parallelized our procedure by dividing up the genome into independent regions, separated by large regions containing zero peak summits (see code for details). Within these, we subdivided regions if peak summits were separated more than 20bp, the resolution of our base signal, and further if the number of peak summits dropped below the median number of peak summits in that region (with a minimum of 1 per base-pair), allowing for the recovery of substructures within tightly clustered DNase I hypersensitive sites. This resulted in a tentative set of isolated accessibility events, anchored on sets of peak summits (**Extended Data Fig. 1b-c, step 4**).

Delineation of consensus DHSs

We assigned genomic coordinates to each isolated event of chromatin accessibility by accumulating contributing peak positions (full start-to-end coordinates) and delineating the Full-Width at Half Maximum (FWHM) on the resulting histogram, representing a consensus across datasets (**Extended Data Fig. 1b-c, step 5**). Starting from local maxima of summit positions, we subsequently included neighboring base-pairs in each direction, up until half of the height at the local maxima. This FWHM approach for

delineating areas of consistent peak regions results in base pair-level delineations supported by at least half of all datasets in which a peak was called at that location.

Each delineation was assigned a consensus summit (centroid) and score (see below). Two delineations were combined in case the summit of one delineation overlaps the coordinates of another delineation with a higher score. We combine delineations by merging their peak summit clusters and re-estimating a single FWHM delineation, to retain high resolution consensus delineations. In a few cases, this step was skipped because it would result in a single delineation where the majority of involved datasets contributed more than one peak (see code for implementation details).

The above resulted in an Index of 3,591,898 DNase I Hypersensitive Sites (DHSs), each occurring across any number of 733 input DNase-seq datasets. We next annotated each individual DHS.

Annotation of individual DHSs

Through the above procedure we obtained perfect provenance of which DNase-seq peaks contribute to each DHS delineation. We used this to estimate the most likely center-of-mass, or centroid, using the median summit position of all contributing peaks. As a measure of dispersion, or positional stability, we further defined a ‘core’ region for each DHS (**Fig. 1c-d, Extended Data Fig. 1e**), consisting of the coordinate range containing 95% of all contributing peak summits, i.e. the 2.5% trimmed range. This is conceptually similar to the interquartile range (IQR), except with a breakdown point of 2.5% instead of 25%. We used this adapted version of the IQR over the standard IQR or confidence interval as the latter two typically resulted in intervals too narrow to meaningfully visualize.

The normalized DNase-seq signal values across datasets provide the basis for per-DHS confidence scores, either summed across all datasets (**Extended Data Fig. 1f**), or by way of the mean signal across summit-contributing datasets (**Extended Data Fig. 1g**).

The former quantifies confidence by way of overall signal level across datasets but may penalize dataset-specific or -restricted DHSs. The latter allows more readily for high-scoring dataset-specific DHSs, but does not directly consider the level of replication across datasets. For most practical purposes we recommend the use of confidence scores based on the mean signal across datasets, along with the number of datasets in which a DHS is found.

Lastly, to facilitate an unambiguous identification of Index DHSs, and accommodate posterity of analyses and results, we propose a flexible naming scheme for individual DHSs. Each DHS was assigned a unique identifier, consisting of the chromosome on which it is located, separated by a period with a number reflecting the approximate chromosomal location percentile (**Fig. 1d**). As such, these identifiers read like floating point numbers, allowing for seamless addition of future newly discovered DHSs in-between existing ones. Additionally, because of this, the length of the identifier reflects the approximate local density of DHSs. These features make this identification system relatively robust to future genome builds and portable to personal genomes. The very nature of the actual identifiers makes them interpretable, as opposed to other databases such as dbSNP (e.g. rs1045642) or Ensembl (e.g. ENSG00000210049). Moreover, the system allows for a direct integration with DNase I Footprint IDs built on DHS IDs (chr.DHS_ID.FP_ID, e.g., footprint 11.149576.2).

Construction of a DHS Vocabulary

Rationale for component-wise description of DHSs

Cluster analysis methods such as hierarchical or k-means clustering have been widely employed to define patterns of gene or regulatory element activity across a range of cell types and states[59,60]. Indeed, hierarchical and partitional clustering has been used to group DHSs with similar behavior across DNase-seq datasets[16,24]. With this approach, each DHS is assigned to a single cluster only, along with other DHSs that display a similar patterning across all datasets. To capture complex patterning of millions of

DHSs across >700 datasets with clustering, thousands of clusters would be required, eclipsing utility and interpretability. This requirement arises because different clusters are in effect capturing incomplete snapshots of the operation of what are in reality a much smaller number of co-occurring biological processes. Moreover, these clusters are unlabeled, each consisting of a complex combination of contributions from different cellular conditions. This makes downstream analyses impractical and does not lead to an improved understanding of the repertoire of regulatory patterns present in the data. As such, clustering is poorly suited to capture the rich spectrum of cross-cell-type DHS activation behavior in a biologically interpretable fashion that is robust and broadly useful for both genome annotation and downstream analytical applications.

Beyond clustering, methods have been proposed to learn chromatin state models from genome-wide data[40,61]. Although primarily designed to model data in a temporal fashion (i.e. along a genome), chromatin state models have been trained jointly across large numbers of cell types in a “stacked” manner in an attempt to model complex inter-cell type patterning[62]. As with clustering, for DNase-seq data across many cell types, this approach typically results in a very large number of chromatin states that cannot be directly interpreted across the context of multiple cell types, requiring post-hoc processing to aid in downstream interpretation.

Here, we decompose the occurrence patterns of DHSs across DNase-seq datasets into a small number of components. By combining components, we can describe the behavior of DHSs across multiple cellular conditions, e.g. a DHS occurring in both neural and immune cell types. This way, a DHS occurring in a single cellular condition may be described by a single component, whereas a DHS occurring in many different cellular conditions may be described by a combination of multiple components. By combining even a modest number of components, with varying relative contributions, we are able to capture a vast combinatorial space.

Decomposition using Non-negative Matrix Factorization (NMF)

Non-negative Matrix Factorization (NMF)[28,29] was used to perform the decomposition of DHS patterns across datasets. Originally introduced in the field of computer vision, in bioinformatics research the major application has so far been to gene expression datasets[63–67]. NMF is distinguished from techniques such as principal component analysis (PCA)[68] or clustering methods that learn holistic rather than parts-based representations of data. NMF aims to model individual ‘parts’ or ‘components’ that in combination describe a larger data pattern or object (e.g., nose, eyes, mouth → face), while holistic methods aim to capture global patterns of variation in data (e.g., shadow patterns over a whole face). An additional advantage of NMF over e.g. PCA, is that due to the non-negativity constraint, the learned components can be combined in an interpretable manner, like ingredients in a recipe.

Briefly, NMF aims to break up an *n*-by-*m* (*n* datasets, *m* DHSs) dimensional matrix **V** into two new matrices **W** (dimensionality *n*-by-*k*) and **H** (dimensionality *k*-by-*m*), the product of which approximately reconstructs the original data (**Extended Data Fig. 3a-c**). NMF is an unsupervised algorithm that reduces the dimensionality of data when *k* is less than both *n* and *m*. In essence, the matrices **W** and **H** provide a reformulation of DNase-seq datasets and DHSs in terms of *k* components, instead of in terms of *m* DHSs or *n* datasets, respectively.

NMF implementation and choice of number of components

We applied NMF decomposition to a binary matrix **V** consisting of presence/absence calls of *m* DHSs across *n* DNase-seq datasets. Using binarized data allowed us to harmonize qualitative differences between promoters and distal elements while approximating the general behavior of distal elements. For a given element, the height of the peak in DNase-seq signal appears to reflect chiefly the population proportion of templates that have adopted an open/remodeled configuration[69]. However, this phenomenon differs qualitatively between promoter vs. distal elements. The former are

larger and more accessible elements; even though they represent the minority of elements, they dominate the top end of the quantitative accessibility landscape. Promoters also exhibit far less cell type-selectivity. By contrast, distal elements, which constitute the vast majority, exhibit considerable lineage- and cell type-selectivity, typically with ‘on/off’ behavior — i.e., most elements are completely ‘off’ in most cell and tissue types.

The number of components *k* to use is a parameter of the NMF algorithm and its choice is a trade-off between how much of the Biological complexity should be captured versus how interpretable the model should be. To systematically choose a good value for *k*, we assessed the quality of the decomposition across a wide range of values using appropriate metrics as follows.

With only 4% non-zero elements, matrix **V** is quite sparse, and thus we employed metrics suitable for highly class-imbalanced and sparse datasets. We calculated precision (TP/(TP+FP)) and recall (TP/(TP+FN)), and subsequently their harmonic average, the F1 score (2 * (precision * recall) / (precision + recall)), utilizing the number of true positives (TP), false positives (FP), and false negatives (FN). In order to assess whether an element in the reconstruction is a positive or negative, we selected a decision boundary which generally maximized total F1 scores across realizations of NMF with various rank *k.* We find that this is the case for a decision boundary of 0.35 (**Extended Data Fig. 3e**).

We considered values of *k* between 4 and 36. While the increase in total F1 scores as a function of *k* is monotonic, the relation is sublinear pointing to diminishing returns (**Extended Data Fig. 3f**). This effect seemed to become prevalent somewhere between 10 and 20 components. Calculating the derivative of the F1 score relative to the number of components *k*, showed that *k*=16 is a point where adding additional components leads to diminishing returns (**Extended Data Fig. 3g**). We therefore chose

to use *k*=16 components for downstream analyses, for a relatively good reconstruction performance while allowing for a simplified lower-dimensional interpretation.

We used the Python scikit-learn package version 0.19.1 implementation of NMF. We used a coordinate descent solver for the Frobenius norm objective function. Non-negative double singular value decomposition (NNDSVD)[48] was used to initialize the weights and induce relative sparsity. A random seed of 20 was used for the SVD algorithm. Other parameters were left as default: tol=0.0001, max_iter=200, alpha=0.0, l1_ratio=0.0. The `umap-learn` Python library was used to embed the NMF decomposition into 2 dimensions, using parameters `min_dist=0.5`, `n_neighbors=200` and `random_state=33` (**Fig. 3d, Extended Data Fig. 10a**).

Regulatory annotation of human genes

Association of genes with DHS components

We do not currently take the magnitude of accessibility of a DHS into consideration in our annotation strategy so as not to overweigh the contributions of typically strong DHSs such as promoters, though this could potentially provide additional richness to the analysis at the cost of increased complexity. We confirmed that the number of component-annotated genes was not biased relative to the number of component-associated biosamples (Spearman rho = 0.13, p = 0.62).

The strength of component association was particularly striking for genes encoding tissue-regulatory factors such as *GATA1* (myeloid/erythroid component DHSs), *FOXP3* (lymphoid component), and *HOXB9* (developmental component). A subset of genes showed enrichment of more than one component suggestive of different functions in different organ systems – for example *CDX2* (embryonic/primitive and digestive components; **Fig. 3c**).

Construction and use of gene expression compendium dataset

To select relevant tissue and cell types for each DHS Vocabulary component we used regular expressions to globally describe and filter on tissue and cell types in the available ARCHS4 dataset metadata, as follows:

Placenta: `placenta|trophoblast`

Lymphoid: `lymph|T-cell|B-cell|CD[48]|regulatory|thymus`

Myeloid/erythroid: `myel|CD34|erythr|cord blood|KBM7|K562`

Cardiac: `cardiac|cardio|heart|ventricle`

Musculoskeletal: `muscle|bone|skeletal|myocyte|tongue EXCEPT marrow|cardio|cardiac`

Vascular/endothelial: `vascul|endotheli|vessel|vein|arter|HMVEC EXCEPT H1`

Embryonic/Primitive: `embryonic|iPSC|H1|H9 EXCEPT derived|fibroblast`

Neural: `neuro|nervous|brain|glia|eye|retina|spinal EXCEPT H1`

Digestive: `digestive|colon|liver|intestin|hepato`

Stromal A & B: `fibroblast EXCEPT iPSC|cardiac`

Renal/cancer: `renal|kidney|podocyte|glomeru|tubular EXCEPT fetal`

Cancer/epithelial: `epithelial|keratino|melanocyt|MCF`

Pulmonary devel.: `fetal AND lung EXCEPT fibroblast`

Renal/organ devel.: `fetal AND kidney`

Datasets occurring in multiple groups, with the exception of Stromal A & B, were removed to prevent ambiguity.

Supplementary Note | DHS Vocabulary component labeling

■**Placental/trophoblast** — Placenta and trophoblast biosamples. Strong enrichment for binding sites of GCM1, a factor selectively expressed in trophoblasts and placenta[70] and associated with pre-eclampsia in pregnant women[71].

■**Embryonic/primitive** — Embryonic stem cells and related biosamples. Most enriched motif is that for POU5F1/OCT4, reflecting a key role in embryonic development and pluripotency.

■**Organ development/renal** — Largely captures fetal kidney biosamples, with strong enrichment for development-related Homeobox protein (HOX) factors, as well as PAX2, associated with early kidney development[72].

■**Pulmonary development** — Consists of fetal lung biosamples. Enriched for CEBPB and FOXC2 motifs, the latter of which is implicated in lung development and maturation[73].

■**Lymphoid** — T-cells and other immune-related cellular conditions. Its most enriched motifs are Interferon-Regulatory Factors (IRF4, IRF1, IRF5, etcetera), in line with their critical role in the (adaptive) immune system[74].

■**Myeloid/erythroid** — CD34+ cells, with strong motif enrichments for not only ETS/SPI1, but also for GATA1 — by itself, as well together with TAL1.

■**Cardiac** — Associated with heart-related biosamples. Strongly enriched for motifs of the Myocyte enhancer factor–2 (MEF2) transcription factor, a core cardiac TF[75].

■**Musculoskeletal** — Associated with muscle(-related) and bone biosamples. Enriched for Musculin (MSC) motifs, with known key roles in regulating myogenesis.

■**Vascular/endothelial** — Consists mostly of HMVEC cells and is enriched for motifs of ERG, a member of the erythroblast transformation-specific (ETS) family of transcription factors, required for platelet adhesion to the subendothelium, inducing vascular cell remodeling.

■**Neural** — Brain and other nervous system biosamples. General enrichment for AT-rich homeobox motifs, as well as NEUROD2 motifs.

■**Digestive** — Associated with intestine, liver and bowel mucosa biosamples. Strong enrichment for motifs of hepatocyte nuclear factor 4 (HNF4), critical for liver development.

■**Renal/cancer** — Mostly adult kidney biosamples, including renal cancer. Enriched for HNF1B, the HNF1A paralog involved in kidney function.

■**Cancer/epithelial** — Associates with various cancer types, as well as general epithelia. The top-scoring motif is for p53 (cancer-related) and p63, which has been proposed to play a dual role[76]: initiating epithelial stratification during development and maintaining proliferative potential of basal keratinocytes in mature epidermis.

■**Stromal A** — Captures fibroblast biosamples. Enriched for motifs of Jun dimerization protein 2 (JDP2), as well as other components of the AP-1 transcription factor (JUN, FOS).

■**Stromal B** — Captures similar Biology as the Stromal A component.

■**Tissue invariant** — No strong association with specific cell and tissue types, but shows enrichment for a diverse set of housekeeping factor motifs, such as CTCF, ETS and NRF.

Supplementary References

59. Eisen, M. B., Spellman, P. T., Brown, P. O. & Botstein, D. Cluster analysis and display of genome-wide expression patterns. *Proc. Natl. Acad. Sci. U. S. A.* **95**, 14863–14868 (1998).
60. D'haeseleer, P. How does gene expression clustering work? *Nat. Biotechnol.* **23**, 1499–1501 (2005).
61. Ernst, J. & Kellis, M. ChromHMM: automating chromatin-state discovery and characterization. *Nat. Methods* **9**, 215–216 (2012).
62. Ernst, J. & Kellis, M. Chromatin-state discovery and genome annotation with ChromHMM. *Nat. Protoc.* **12**, 2478–2492 (2017).
63. Devarajan, K. Nonnegative matrix factorization: an analytical and interpretive tool in computational biology. *PLoS Comput. Biol.* **4**, e1000029 (2008).
64. Ochs, M. F. & Fertig, E. J. Matrix factorization for transcriptional regulatory network inference. in *2012 IEEE Symposium on Computational Intelligence in Bioinformatics and Computational Biology (CIBCB)* 387–396 (2012).
65. Stein-O'Brien, G. L. *et al.* PatternMarkers & GWCoGAPS for novel data-driven biomarkers via whole transcriptome NMF. *Bioinformatics* **33**, 1892–1894 (2017).
66. Kim, J. W. *et al.* Decomposing Oncogenic Transcriptional Signatures to Generate Maps of Divergent Cellular States. *Cell Syst* **5**, 105–118.e9 (2017).
67. Alexandrov, L. B. *et al.* Signatures of mutational processes in human cancer. *Nature* **500**, 415–421 (2013).
68. Hotelling, H. Analysis of a complex of statistical variables into principal

components. *Journal of Educational Psychology* vol. 24 498–520 (1933).

69. Stergachis, A. B., Debo, B. M., Haugen, E., Stirling Churchman, L. & Stamatoyannopoulos, J. A. Single-molecule regulatory architectures captured by chromatin fiber sequencing. *Science* **368**, 1449–1454 (2020).

70. Chen, C.-P., Chen, C.-Y., Yang, Y.-C., Su, T.-H. & Chen, H. Decreased placental GCM1 (glial cells missing) gene expression in pre-eclampsia. *Placenta* **25**, 413–421 (2004).

71. Bainbridge, S. A. *et al.* Effects of reduced Gcm1 expression on trophoblast morphology, fetoplacental vascularity, and pregnancy outcomes in mice. *Hypertension* **59**, 732–739 (2012).

72. Dressler, G. R. Patterning and early cell lineage decisions in the developing kidney: the role of Pax genes. *Pediatr. Nephrol.* **26**, 1387–1394 (2011).

73. Tsuji, M. *et al.* The role of Foxc2 gene in lung development. *Eur. Respir. J.* **44**, P829 (2014).

74. Yanai, H., Negishi, H. & Taniguchi, T. The IRF family of transcription factors: Inception, impact and implications in oncogenesis. *Oncoimmunology* **1**, 1376–1386 (2012).

75. Desjardins, C. A. & Naya, F. J. The Function of the MEF2 Family of Transcription Factors in Cardiac Development, Cardiogenomics, and Direct Reprogramming. *J Cardiovasc Dev Dis* **3**, (2016).

76. Koster, M. I., Kim, S., Mills, A. A., DeMayo, F. J. & Roop, D. R. p63 is the molecular switch for initiation of an epithelial stratification program. *Genes Dev.* **18**, 126–131

(2004).
