# Supplementary material for: Index and biological spectrum of human DNase I hypersensitive sites
Source: Nature. 2020 Jul 29;584(7820):244–51. doi: 10.1038/s41586-020-2559-3 (PMC7422677; doi:10.1038/s41586-020-2559-3)
Supplement: Supplementary file 2 — Reporting Summary [file 41586_2020_2559_MOESM2_ESM.pdf]

nature research

Corresponding author(s): Wouter Meuleman, John Stamatoyannopoulos

Last updated by author(s): Jun 28, 2020

Reporting Summary

Nature Research wishes to improve the reproducibility of the work that we publish. This form provides structure for consistency and transparency in reporting. For further information on Nature Research policies, see our Editorial Policies and the Editorial Policy Checklist.

Statistics

For all statistical analyses, confirm that the following items are present in the figure legend, table legend, main text, or Methods section.

n/a	Confirmed	
☐	☒	The exact sample size (*n*) for each experimental group/condition, given as a discrete number and unit of measurement
☐	☒	A statement on whether measurements were taken from distinct samples or whether the same sample was measured repeatedly
☐	☒	The statistical test(s) used AND whether they are one- or two-sided<br>*Only common tests should be described solely by name; describe more complex techniques in the Methods section.*
☐	☒	A description of all covariates tested
☐	☒	A description of any assumptions or corrections, such as tests of normality and adjustment for multiple comparisons
☐	☒	A full description of the statistical parameters including central tendency (e.g. means) or other basic estimates (e.g. regression coefficient) AND variation (e.g. standard deviation) or associated estimates of uncertainty (e.g. confidence intervals)
☐	☒	For null hypothesis testing, the test statistic (e.g. *F*, *t*, *r*) with confidence intervals, effect sizes, degrees of freedom and *P* value noted<br>*Give P values as exact values whenever suitable.*
☒	☐	For Bayesian analysis, information on the choice of priors and Markov chain Monte Carlo settings
☒	☐	For hierarchical and complex designs, identification of the appropriate level for tests and full reporting of outcomes
☒	☐	Estimates of effect sizes (e.g. Cohen's *d*, Pearson's *r*), indicating how they were calculated

Our web collection on statistics for biologists contains articles on many of the points above.

Software and code

Policy information about availability of computer code

Data collection	Data collection was assisted by the following pieces of software for read mapping and subsequent primary data processing: BWA (0.7.12), bedops (2.4.39), hotspot2 (2.1.1). All data collection and processing procedures are documented as part of the ENCODE DCC pipelines.
Data analysis	Data analysis was performed using R (version 3.6.1, using packages qvalue (v2.18.0), Matrix (v1.2.17), gplots (v3.0.3), Rtsne (v0.15)) and Python (version 3.6.4, using libraries numpy (v1.18.1), pandas (v1.0.3), sklearn (for NMF, v0.22.1), umap (v0.4.1)). Code is available on Github for building the index of consensus DHSs (https://github.com/Altius/Index), for constructing the DHS vocabulary and the addition of novel biosamples (https://github.com/Altius/Vocabulary), and for annotating DHSs relative to genes and repetitive elements (https://github.com/Altius/DHS_Annotations).

For manuscripts utilizing custom algorithms or software that are central to the research but not yet described in published literature, software must be made available to editors and reviewers. We strongly encourage code deposition in a community repository (e.g. GitHub). See the Nature Research guidelines for submitting code & software for further information.

Data

Policy information about availability of data

All manuscripts must include a data availability statement. This statement should provide the following information, where applicable:

- Accession codes, unique identifiers, or web links for publicly available datasets
- A list of figures that have associated raw data
- A description of any restrictions on data availability

All primary data are available from the ENCODE DCC portal. Biosample metadata are available in Supplementary Table 1 as well as in other formats via Zenodo (https://doi.org/10.5281/zenodo.3838751). The set of 3.5M+ DHS delineations is available in tab-separated format from the ENCODE DCC portal (https://www.encodeproject.org/annotations/ENCSR857UZV/) and via Zenodo (https://doi.org/10.5281/zenodo.3838751). Data matrices describing the occurrence

patterns of DHSs across biosamples are available in various formats via Zenodo (https://doi.org/10.5281/zenodo.3838751). There are no restrictions on data availability and (re)use. We additionally provide a specialized data browser (https://index.altius.org/) and a trackhub for the UCSC Genome Browser (https://genome.ucsc.edu/cgi-bin/hgTracks?db=hg38&hubUrl=https://resources.altius.org/~meuleman/DHS_Index_tracks/hub.txt). BED files documenting the coordinates and annotations of DHSs with evidence of being bound by specific transcription factors are available via Zenodo (https://doi.org/10.5281/zenodo.3838751), and top-scoring elements per TF can be explored in a browser (https://index.altius.org/?application=viewer&roiSet=TFassoc_Meuleman). Various databases were consulted to obtain gene expression data (ARCHS4, https://amp.pharm.mssm.edu/archs4/download.html, accessed June 26, 2018), gene annotations (GENCODE v28 https://www.gencodegenes.org/human/release_28.html, psiCube http://pseudogene.org/psicube/data/gencode.v10.pgene.parents.txt) and pathway annotations (MSigDB v6.1 https://www.gsea-msigdb.org/gsea/msigdb/collections.jsp, KEGG v89.1 https://www.genome.jp/kegg/).

Field-specific reporting

Please select the one below that is the best fit for your research. If you are not sure, read the appropriate sections before making your selection.

☒ Life sciences ☐ Behavioural & social sciences ☐ Ecological, evolutionary & environmental sciences

For a reference copy of the document with all sections, see nature.com/documents/nr-reporting-summary-flat.pdf

Life sciences study design

All studies must disclose on these points even when the disclosure is negative.

Sample size	We determined sample size by considering exclusively high quality DNase-seq data with a SPOT score of at least 0.3 for our analyses, yielding a total of 733 DNase-seq datasets. Collectively, these data assay a total of 438 cell and tissue states spanning all human organ systems (Supplementary Table 1). This is currently the largest high-quality DNase I chromatin accessibility dataset available and as such its sample size is quite adequate.
Data exclusions	We removed DNase-seq samples with a SPOT score below 0.3, to ensure we only retain high quality data (Supplementary Table 1; Google Spreadsheet).
Replication	As part of the ENCODE data collection procedure, we have performed second replicate assays when enough material was available, and have retained replicates for analysis in case SPOT scores were at least 0.3, which is the case for 169 cell types and states. We do not have information on cases where our strict quality metrics were not achieved.
Randomization	Not applicable, as we are not performing any group-wise / cohort-wise comparisons.
Blinding	Not applicable, individual biosample data was obtained and jointly processed and analyzed.

Reporting for specific materials, systems and methods

We require information from authors about some types of materials, experimental systems and methods used in many studies. Here, indicate whether each material, system or method listed is relevant to your study. If you are not sure if a list item applies to your research, read the appropriate section before selecting a response.

Materials & experimental systems

n/a	Involved in the study	
☒	☐	Antibodies
☐	☒	Eukaryotic cell lines
☒	☐	Palaeontology and archaeology
☒	☐	Animals and other organisms
☐	☒	Human research participants
☒	☐	Clinical data
☒	☐	Dual use research of concern

Methods

n/a	Involved in the study	
☒	☐	ChIP-seq
☒	☐	Flow cytometry
☒	☐	MRI-based neuroimaging

Eukaryotic cell lines

Policy information about cell lines

Cell line source(s)	The source of all cell line material is described (when available) in Supplementary Table 1 and the online Google Spreadsheet. Cell lines were procured from appropriate commercial sources. h.ESC lines used were from NIH approved list and provided by laboratories with expertise in growing, characterizing and differentiating these cell types. (see ENCODE website for details and protocols).
Authentication	Authentication was provided by the commercial vendors from which the cell lines were procured and in accordance with ENCODE policies. Beyond this, no cell lines were further authenticated.
Mycoplasma contamination	Cell lines were not routinely checked for mycoplasma contamination.
Commonly misidentified lines (See ICLAC register)	One included cell line (SK-N-MC) appears on the ICLAC list, and we properly document this biosample as Ewing's sarcoma as opposed to a neuroepithelioma biosample.

Human research participants

Policy information about studies involving human research participants

Population characteristics	Population characteristics and other biosample metadata are described (when available) in Supplementary Table 1 and the online Google Spreadsheet. Human primary tissues were collected under IRB approved protocols using Open-access consents, as per NHGRI/ENCODE policies. Care was taken to ensure sex parity for tissues collected, to the extent possible. All primary tissues processed by our center were received as anonymized samples with minimal metadata information about tissue type, age, sex and ethnicity of the donor. As such, our institutional IRB reviewed our study using anonymized primary tissue and cell samples, and classified it as non-human subject studies.
Recruitment	Donors were recruited by collection centers (like Fred Hutch and dbGaP), using IRB approved Open-access consents. We received anonymized
Ethics oversight	University of Washington IRB

Note that full information on the approval of the study protocol must also be provided in the manuscript.
